# Post-extinction recovery of the Phanerozoic oceans and biodiversity hotspots

**DOI:** 10.1038/s41586-022-04932-6

**Published:** 2022-07-13

**Authors:** Pedro Cermeño, Carmen García-Comas, Alexandre Pohl, Simon Williams, Michael J. Benton, Chhaya Chaudhary, Guillaume Le Gland, R. Dietmar Müller, Andy Ridgwell, Sergio M. Vallina

**Affiliations:** 1 Institut de Ciències del Mar, Consejo Superior de Investigaciones Científicas, Barcelona, Spain; 2 Department of Earth and Planetary Sciences, University of California, Riverside, Riverside, CA USA; 3 Biogéosciences, UMR 6282, UBFC/CNRS, Université Bourgogne Franche-Comté, Dijon, France; 4 State Key Laboratory of Continental Dynamics, Department of Geology, Northwest University, Xi’an, China; 5 EarthByte Group, School of Geosciences, University of Sydney, Sydney, New South Wales Australia; 6 School of Earth Sciences, University of Bristol, Bristol, UK; 7 Alfred Wegener Institute, Helmholtz Centre for Polar and Marine Research, Bremerhaven, Germany; 8 Instituto Español de Oceanografía, Consejo Superior de Investigaciones Científicas, Gijón, Spain

**Keywords:** Palaeontology, Evolutionary ecology, Palaeoecology, Evolutionary ecology

## Abstract

The fossil record of marine invertebrates has long fuelled the debate as to whether or not there are limits to global diversity in the sea^1–5^. Ecological theory states that, as diversity grows and ecological niches are filled, the strengthening of biological interactions imposes limits on diversity^6,7^. However, the extent to which biological interactions have constrained the growth of diversity over evolutionary time remains an open question^1–5,8–11^. Here we present a regional diversification model that reproduces the main Phanerozoic eon trends in the global diversity of marine invertebrates after imposing mass extinctions. We find that the dynamics of global diversity are best described by a diversification model that operates widely within the exponential growth regime of a logistic function. A spatially resolved analysis of the ratio of diversity to carrying capacity reveals that less than 2% of the global flooded continental area throughout the Phanerozoic exhibits diversity levels approaching ecological saturation. We attribute the overall increase in global diversity during the Late Mesozoic and Cenozoic eras to the development of diversity hotspots under prolonged conditions of Earth system stability and maximum continental fragmentation. We call this the ‘diversity hotspots hypothesis’, which we propose as a non-mutually exclusive alternative to the hypothesis that the Mesozoic marine revolution led this macroevolutionary trend^12,13^.

## Abstract (web summary)

The diversity hotspots hypothesis attributes the overall increase in global diversity during the Late Mesozoic and Cenozoic eras to the development of diversity hotspots under prolonged conditions of Earth system stability and maximum continental fragmentation.

## Main

The question of whether or not there is an equilibrium diversity that the biota, or portions of the biota, cannot exceed has led to decades of debate between those who think that there is a limit to the global diversity that the Earth can carry^2,3,10^ (that is, a carrying capacity) and those who think that the biosphere is so far from the equilibrium diversity (that is, its carrying capacity) that we can ignore the existence of any limit^4,5,11^. This question has traditionally been addressed by examining the shape of global fossil diversity curves^3,14^. For example, the Palaeozoic era plateau in marine invertebrate diversity is generally taken as strong evidence for the existence of ecological limits to further diversification^3,15^. However, as diversity varies considerably among geographical regions, and each geographical region has its own geological and environmental history, addressing this question requires simultaneously reconstructing the dynamics of regional diversity in both space and time^16,17^. If diversity dynamics were governed by diversity-dependent feedbacks on speciation and extinction rates, then regional diversity should remain stable regardless of time once the carrying capacity has been reached (that is, the logistic model). By contrast, if evolutionary rates were independent of standing diversities, then we should observe positive relationships between evolutionary time-within-regions (or time-for-speciation) and diversity; the older the habitat, the longer the lineages have had to diversify and fill empty niches or explore new ones (that is, the exponential model). Determining which diversification model best describes the dynamics of regional diversity is key to understanding the mechanisms that underlie biogeographical patterns and macroevolutionary trends. However, the fossil record is biased by uneven geographical and stratigraphic sampling efforts^17,18^ and variation in the rock record available for sampling^19^, hindering our ability to investigate the effect of geographical variability in evolutionary time and diversification rate.

To overcome this limitation, we coupled two alternative models of diversification—logistic and exponential—to a global model of palaeogeography and plate-motion that constrains evolutionary time-within-regions. In both diversification models, the net diversification rate varies within a fixed range of values as a function of seawater temperature and food supply, which are reconstructed using a spatially explicit palaeo–Earth system model (Methods and Extended Data Table 1). In the logistic model, the spatially resolved effective carrying capacities (*K*_eff_) are allowed to vary within a fixed range of values (from *K*_min_ to *K*_max_) as a positive linear function of food availability in each ocean region and time. We set relatively low *K*_min_ and *K*_max_ values to enforce diversity saturation, hereafter referred to as the ‘saturated’ logistic model. Mass extinctions are imposed by imputing negative diversification rates to regional communities and assuming non-selective extinction. The percentage of diversity loss as well as the starting time and duration of mass extinctions were extracted from three fossil diversity curves of reference^20–22^.

## Reconstructing global diversity dynamics

**Fig. 1:**
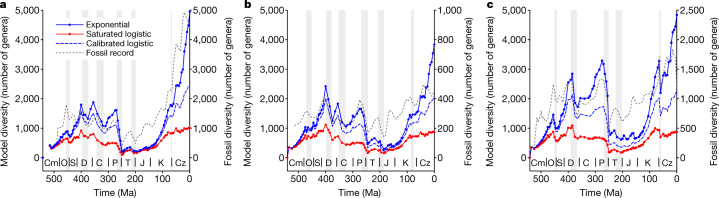
Global diversity dynamics during the Phanerozoic. **a**–**c**, Global diversity dynamics reconstructed from the saturated logistic model (red), the exponential model (blue) and the calibrated logistic model (blue dashed line) after imposing the pattern of mass extinctions extracted from the fossil diversity curve of ref. ^20^ (**a**), ref. ^21^ (**b**) and ref. ^22^. (**c**). In each panel, the corresponding fossil diversity curve is superimposed (grey). The shaded areas represent mass extinctions. C, Carboniferous; Cm, Cambrian; Cz, Cenozoic; D, Devonian; J, Jurassic; K, Cretaceous; O, Ordovician; P, Permain; S, Silurian; T, Triassic. Source data

**Extended Data Fig. 1:**
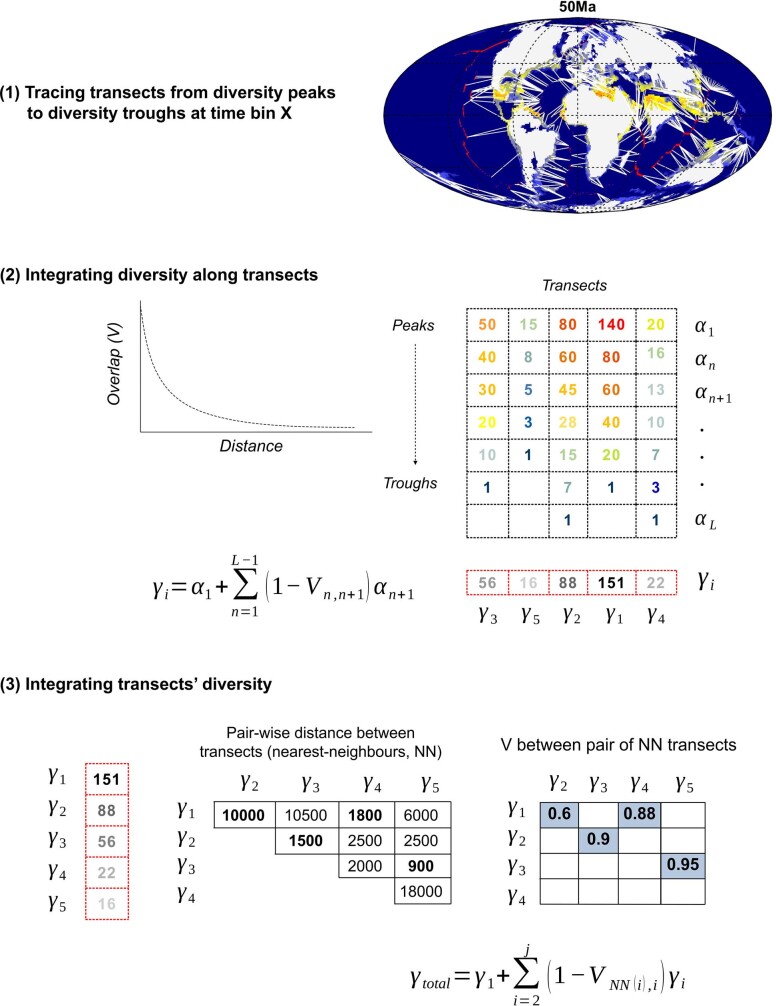
Computing global diversity from diversity maps. For each time interval or regional diversity map, (1) we plot hundreds of transects (white lines) from diversity peaks to their nearest troughs (Supplementary Video 7 for an example), (2) we integrate diversity along the transects (from α_ð‘⟩_ to α_L_) according to the distance between pairs of grids using the overlap coefficient (V), which gives the proportion of shared genera with respect to the grid with the least diversity, i.e. *V*_n, n+1 _× min(α_n_; α_n+1_) [**see** Supplementary Note 1**in SI**], (3) we order the resulting transects’ diversity (γ_i_) from maximum diversity (γ_max_) to minimum diversity (γ_min_), calculate the pair-wise distance between transects (the distance between their peaks), and integrate the diversity of transects from the greatest to lowest according to the nearest-neighbour distance of the corresponding transect to those transects already integrated (γ_total_).

**Extended Data Fig. 2:**
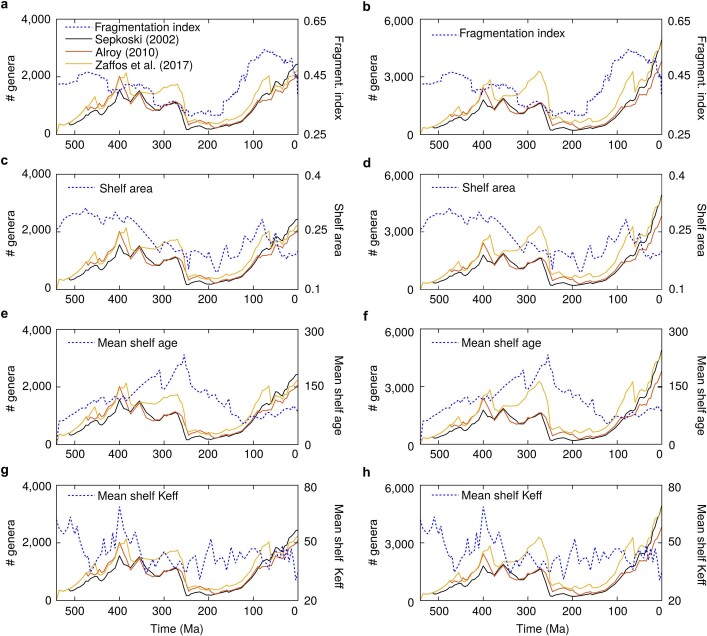
Global diversity dynamics and continental configuration. Global diversity dynamics (# genera) reconstructed by using the calibrated logistic model (**a**, **c**, **e**, **g**) and the exponential model (**b**, **d**, **f**, **h**) and overimposed on the temporal variability of the fragmentation index **a**, (**a, b**), global mean shelf area (**c**, **d**), mean shelf age (**e**, **f**) and mean shelf effective carrying capacity (K_eff_) (**g**, **h**).

**Extended Data Fig. 3:**
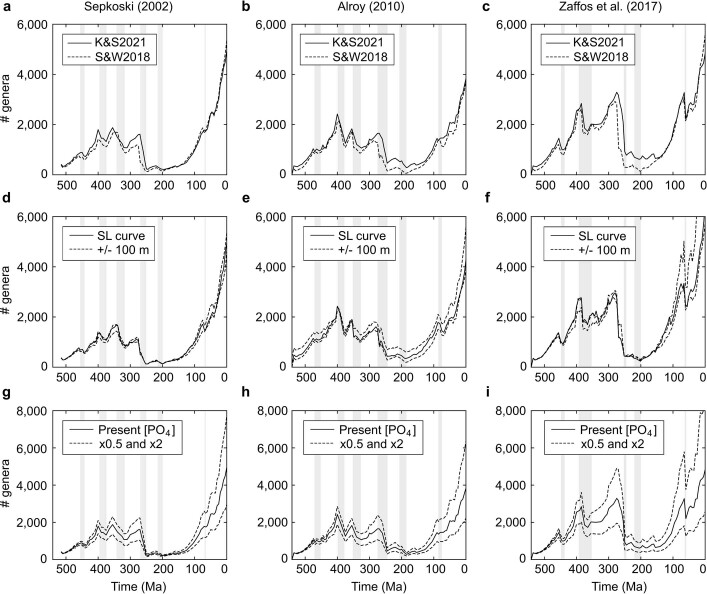
Sensitivity analysis. **a-c**, Global diversity dynamics reconstructed by the exponential diversification model using two alternative palaeogeographic models^33,35^. **d-f**, Effect of changing the sea level +/− 100 m compared to the original palaeo-digital elevation model of Scotese and Wright^33^. **g-i**, Effect of changing the mean ocean phosphate concentration (x2 and x0.5 with respect to present day concentrations, i.e., default scenario). All simulations were run for each of the three mass extinction patterns, that is, Sepkoski^20^ (**a**, **d**, **g**), Alroy^21^ (**b**, **e**, **h**), and Zaffos et al^22^. (**c**, **f**, **i**).

Each of the two diversification models tested here produces a total of 82 spatially explicit reconstructions of diversity spanning from the Cambrian period (541 million years ago (Ma)) to the present (Supplementary Videos 1 and 2). On each of the 82 diversity distribution maps, we traced hundreds of line transects from diversity peaks to their nearest diversity troughs and integrated the total diversity in each transect by assuming a decay function in taxonomic similarity with geographical distance (Methods and Extended Data Fig. 1). For each of the 82 time intervals, all integrated diversities along transects were then reintegrated stepwise, from the transect with the greatest diversity to the transect with the lowest one, assuming the same distance-decay function applied to individual transects. The resulting global diversity estimates were plotted against the midpoint value of the corresponding time interval to generate a synthetic global diversity curve. Both the saturated logistic model and the exponential model produced relatively similar global diversity dynamics (Fig. 1). This is to be expected as the global diversity curves produced by both models were equally influenced by mass extinctions and long-term variations in the degree of continental fragmentation (Extended Data Fig. 2). However, although both models show similar diversity dynamics, the amplitudes of global diversity variations differ markedly between models (Fig. 1). The exponential model gives rise to conspicuous increases in global diversity from the Cambrian to Late Ordovician period, the Silurian period to Early Devonian period, the Carboniferous period (Early to Late Pennsylvanian subperiod), the Early to Late Cretaceous period and the Palaeocene epoch to the present. The Permian–Triassic period mass-extinction event lowered global diversity to Early Palaeozoic levels, but later diversification led Late Cretaceous and Neogene period faunas to exceed the mid-Palaeozoic global diversity peak. These trends emerge consistently regardless of which mass extinction pattern was imposed^20–22^ (Fig. 1). The logistic model also reproduces the initial increase in diversity, from the Cambrian to Late Ordovician and from the Silurian to Early Devonian (Fig. 1). However, in contrast to the exponential model, in the saturated logistic model, this initial upwards trend is followed by a convex diversity pattern that is interrupted by a modest increase during the Cretaceous, which rarely exceeds the mid-Palaeozoic global diversity peak in our set of simulations. A sensitivity analysis revealed that these global dynamics are barely affected by (1) a sea level rise/fall of 100 m relative to the sea level of the original palaeo–digital elevation model and (2) halving or doubling the mean ocean phosphate concentration, to decrease/increase food availability (Extended Data Fig. 3).

## The calibrated logistic model

**Extended Data Fig. 4:**
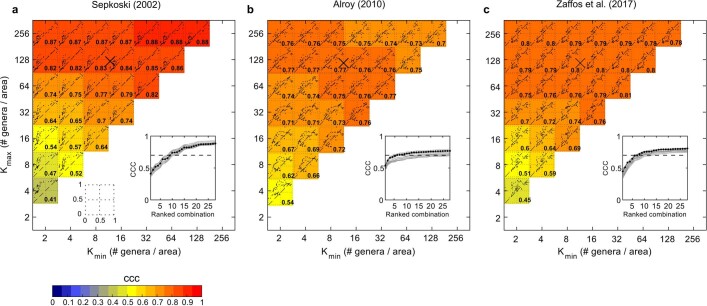
Calibrating the logistic model’s carrying capacities. **a-c**, Lin’s concordance correlation coefficient (CCC) for the relationship between the global diversities resulting from the model and the fossil diversity estimates of Sepkoski^20^ (n = 66 data points) (**a**), Alroy^21^ (n = 60) (**b**), and Zaffos et al^22^ (n = 69) (**c**) using different combinations of *K*_min_ and *K*_max_ in the model. Only the time series data between the end of one mass extinction and the beginning of the next were included in the analysis .Superimposed on each combination of *K*_min_ and *K*_max_ are the scatterplots of the normalized (0-1) diversity data (model vs fossil). The inset in each panel shows the CCCs in ascending order for the different combinations of *K*_min_ and *K*_max_. The black curve in the insets is for the simulation run using the selected parameters [i.e., *Q*_10_ = 1.75, *K*_food_ = 0.5 molC m^−2^y^−1^, net diversification rate limits (*ρ*_min_ − *ρ*_max_) = 0.001-0.035 Myr^−1^]. The grey curves are for each of the first 15 combinations of parameters listed in Extended Data Table 2. The dashed line denotes the CCC value of 0.7 and the cross in each panel is the average of all *K*_min_ and *K*_max_ combinations giving a CCC greater than 0.7. These are the values of *K*_min_ and *K*_max_ used to run the calibrated logistic model.

All other things being equal, the higher the *K*_min_ and *K*_max_ values of the logistic model, the longer the time required to reach diversity saturation. Consequently, the choice of *K*_min_ and *K*_max_ critically influences the extent to which regional biotas reach saturation. To calibrate the *K*_min_ and *K*_max_ parameters, we run simulations of pairwise *K*_min_ and *K*_max_ combinations in a geometric sequence of base 2, from 2 to 256 genera, and tested the effect of changing the *K*_min_ and *K*_max_ values on the concordance between the normalized global diversities generated by the model and those estimated from the fossil record. We focused the analysis on the time-series data between the end of one mass extinction and the beginning of the next, that is, considering those time intervals dominated by rising diversity trajectories. Lin’s concordance correlation coefficient (CCC) increases with increasing *K*_min_ and *K*_max_ until reaching a plateau, except for the mass extinction pattern of Sepkoski^20^, for which it continues to increase even at the highest *K*_min_ and *K*_max_ values (Extended Data Fig. 4). These results were consistently replicated using alternative values for the parameters of the model that define the temperature and food dependence of the net diversification rate (Extended Data Fig. 4 (insets, grey lines) and Extended Data Table 2).

**Fig. 2:**
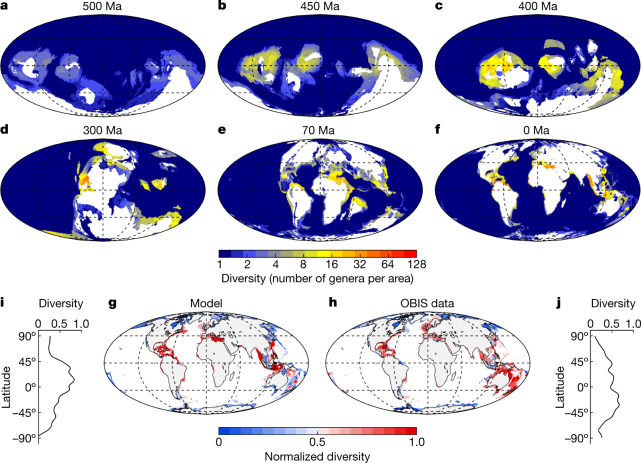
Re-diversifying the Phanerozoic oceans. **a**–**f**, Spatial distribution of marine animal diversity (number of genera per area) in the Cambrian (Guzhangian, 500 million years ago (Ma); **a**), Late Ordovician (Katian, 450 Ma; **b**), Early Devonian (Emsian, 400 Ma; **c**), Late Carboniferous (Pennsylvanian, 300 Ma; **d**), Late Cretaceous (Maastrichtian, 70 Ma; **e**) and the present day (**f**) generated by the calibrated logistic model after imposing the pattern of mass extinctions extracted from the fossil diversity curve of ref. ^20^. This model run uses the following parameters: *Q*_10_ = 1.75, *K*_food_ = 0.5 molC m^−2^ yr^−1^, net diversification rate limits (*ρ*_min_ − *ρ*_max_) = 0.001–0.035 Myr^−1^ (per capita), and a *K*_min_ to *K*_max_ range between 12 and 123 genera per area according to the calibration analysis presented in Extended Data Fig. 4. The same plots but for the mass extinction patterns extracted from the fossil diversity curves of refs. ^21,22^ are shown in Extended Data Fig. 5. The full Phanerozoic sequences are shown in Supplementary Video 3. **g**,**h**, Current spatial distributions of diversity along the continental margins from model simulations (**g**) and field observations extracted from the OBIS database (genera belonging to subphylum Crustacea and phylum Mollusca) (**h**). For the purpose of comparison, normalized diversities (0–1) bounded between quantiles 0.05 and 0.95 are represented. **i**,**j**, Zonal mean diversity for 20° latitudinal bands from model simulations (**i**) and field observations extracted from the OBIS database (**j**). Source data

**Extended Data Fig. 5:**
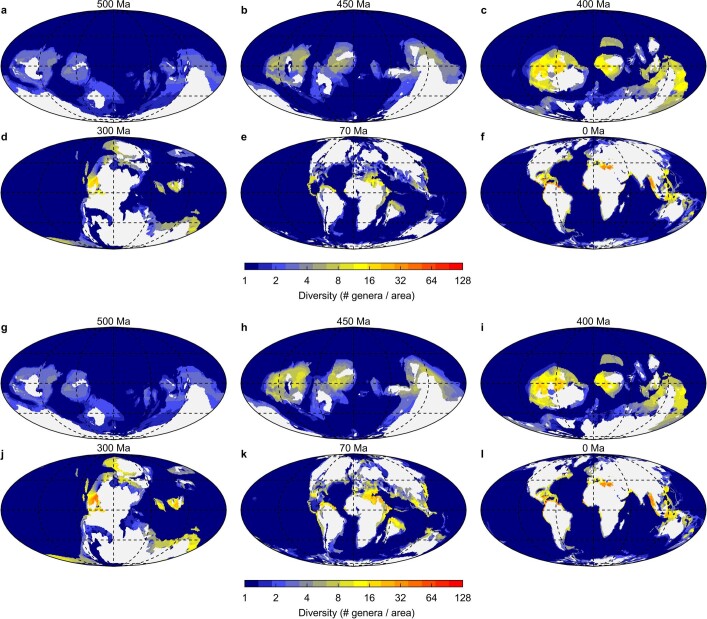
Re-diversifying the Phanerozoic oceans. **a-f**, Spatial distribution of marine animal diversity (# genera/area) in the Cambrian (Guzhangian, 500 Ma), Late Ordovician (Katian, 450 Ma), Early Devonian (Emsian, 400 Ma), Late Carboniferous (Pennsylvanian, 300 Ma), Late Cretaceous (Maastrichtian, 70 Ma) and present generated by the calibrated logistic model after imposing the pattern of mass extinctions extracted from the fossil diversity curve of Alroy^21^. **g-l**, as in a-f but for the pattern of mass extinctions extracted from the fossil diversity curve of Zaffos et al^22^. These model runs use the following parameters: *Q*_10_ = 1.75, *K*_food_ = 0.5 molC m^−2^y^−1^, net diversification rate limits (*ρ*_min_ − *ρ*_max_) = 0.001-0.035 Myr^−1^ (per capita), and a *K*_min_ to *K*_max_ range between 11 and 119 genera per unit area for Alroy^21^ and between 11 and 117 genera for Zaffos et al^22^. See also Supplementary Video 3 for the full Phanerozoic sequences.

**Extended Data Fig. 6:**
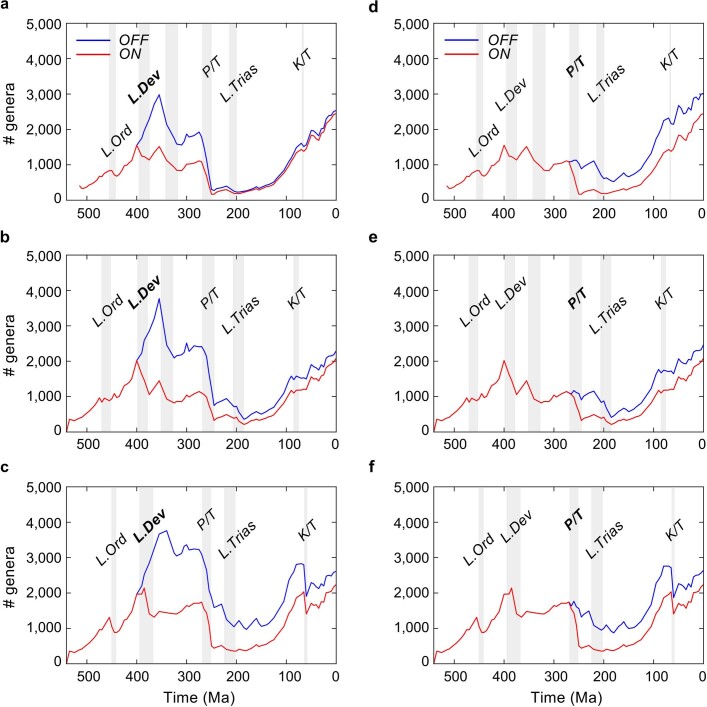
Testing the effect of mass extinctions. Global diversity dynamics (# genera) reconstructed by the calibrated logistic model after disabling (blue) and enabling (red) the Late Devonian (**a-c**) and Permo-Triassic (**d-f**) mass extinctions from the mass extinction pattern extracted from the fossil diversity curve of Sepkoski^20^ (**a**, **d**), Alroy^21^ (**a**, **e**) and Zaffos et al^22^ (**a**, **f**), respectively.

We next re-run the logistic model using the average of all *K*_min_ and *K*_max_ combinations giving a CCC greater than 0.70, hereafter referred to as the ‘calibrated’ logistic model. The calibrated model generates global diversity curves half way between the two end-member diversification models—the saturated logistic and the exponential (Fig. 1). Most of the diversity is concentrated in shallow marine environments, in which high temperatures and abundant food supplies increase the rates of diversification compared with the deep-sea habitats (Fig. 2a–f and Extended Data Fig. 5). Diversity hotspots occur in tropical shelf seas of the Early Devonian, Permian, Late Cretaceous and Cenozoic (Fig. 2c–f and Supplementary Video 3). During the Early Devonian, diversity hotspots developed on the western continental margins of Laurentia and Siberia as well as on the tropical shelves of Gondwana. The recovery of Laurentian diversity hotspots after the Late Devonian mass extinction led to the onset of Permian hotspots, which eventually disappeared during the Permian–Triassic mass extinction. Diversity hotspots became particularly prominent during the Late Cretaceous and Cenozoic in the western basins of the Tethys Ocean, the Arabian Peninsula, the Atlantic Caribbean-East Pacific and the Indo-West Pacific provinces (Fig. 2e,f). This temporal trend in the prominence of diversity hotspots cannot be explained by a secular increase in the maximum lifetime of shelf seas. Geological data from ancient continental margins trapped within orogenic belts^23^ and global tectonic reconstructions^24^ show no evidence of an increase in the lifespan of passive continental margins or in the maximum ages of the seafloor over the Phanerozoic. Rather, we argue that the temporal proximity between the Ordovician–Silurian (Hirnantian stage), Late Devonian (Frasnian–Famennian stage) and Permian–Triassic mass extinctions, coinciding with a long-lived phase of marine shelves destruction during the assembly of Pangaea, interrupted the development of diversity hotspots during the Palaeozoic. In fact, by deactivating the Late Devonian mass extinction in the model, we found that the development of diversity hotspots before the end of the Permian leads to global diversities that are two to three times greater than those generated by the same calibrated logistic model with all mass extinctions enabled (Extended Data Fig. 6a–c). By contrast, the comparatively long expanse of time that separated the mass extinctions of the end-Triassic and end-Cretaceous extended the time-for-speciation under conditions of increasing continental fragmentation, giving rise to exceptionally high-diversity regions before the Cretaceous–Palaeogene mass extinction. The extraordinary diversity of Late Cretaceous hotspots ensured the continuity of relatively high diversity levels in the aftermath of the end-Cretaceous mass extinction, facilitating the subsequent development of diversity hotspots during the Cenozoic.

To evaluate the performance of the model in reconstructing the spatial distributions of diversity, we compared the results of the present-day calibrated logistic model (that is, 0 Ma) with observations of two of the most relevant groups of marine invertebrates, crustaceans and molluscs, extracted from the Ocean Biodiversity Information System (OBIS)—a global database of occurrence records of marine taxa (Methods). The regional diversity map generated by the model shows reasonable similarities to the observed diversity distributions along the continental margins (Fig. 2g,h). The main discrepancies between the model and the OBIS data occur in the surroundings of Australia and New Zealand, where the model underestimates diversity. The model lacks long-distance dispersal, which precludes a more detailed reconstruction of the spatial structuring of diversity in such a highly interconnected ocean region. On the other hand, the OBIS data are not homogeneously distributed over the global ocean and, although our analysis attempts to minimize this bias (Methods), some of the discrepancies between model and observations may be due to database limitations. Despite these discrepancies, both observed and modelled diversity decline from the equator towards the poles (Fig. 2i,j), with most diversity concentrated in the Indo-West Pacific, the Atlantic Caribbean-East Pacific and the Mediterranean (Fig. 2g,h).

**Fig. 3:**
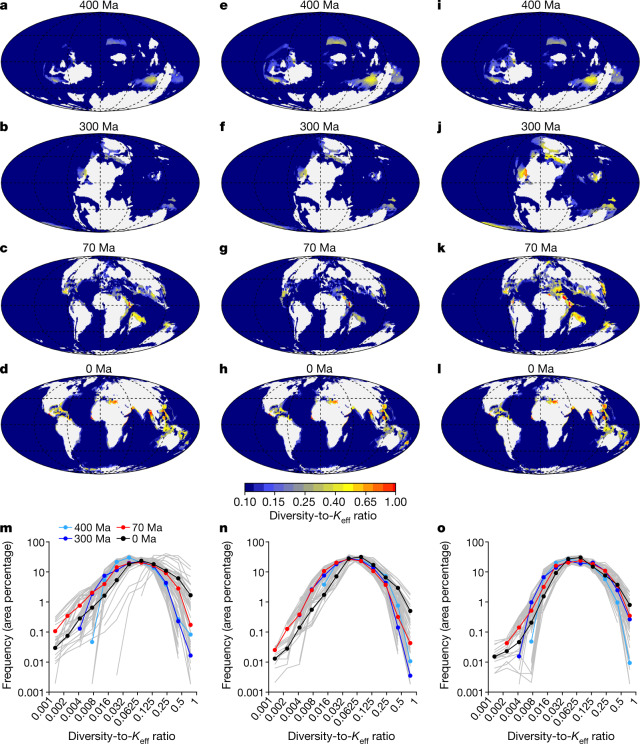
The pervasiveness of ecological unsaturation. **a**–**l**, Spatial distribution of the diversity-to-*K*_eff_ ratio in deep-sea habitats and flooded continental regions of the Early Devonian (Emsian, 400 Ma; **a**,**e**,**i**), Late Carboniferous (Pennsylvanian, 300 Ma; **b**,**f**,**j**), Late Cretaceous (Maastrichtian, 70 Ma; **c**,**g**,**k**) and the present day (**d**,**h**,**l**) generated by the calibrated logistic model after imposing the pattern of mass extinctions extracted from the fossil diversity curve of ref. ^20^ (**a**–**d**), ref. ^21^ (**e**–**h**) and ref. ^22^ (**i**–**l**). The full Phanerozoic sequences are shown in Supplementary Video 4. **m**–**o**, Frequency distributions (area percentage) of the diversity-to-*K*_eff_ ratio for the flooded continental regions generated by the calibrated logistic model after imposing the pattern of mass extinctions extracted from the fossil diversity curve of ref. ^20^ (**m**), ref. ^21^ (**n**) and ref. ^22^ (**o**). The grey lines are the frequency distributions generated from model simulations using the first 15 parameter settings listed in Extended Data Table 2. The coloured dots are average values for different Phanerozoic times.

Using the outputs of the calibrated logistic model, we analysed the spatial and temporal variability of the diversity-to-*K*_eff_ ratio. This ratio provides a quantitative index of how far (ratios close to zero) or how close (ratios close to one) the regional faunas are from noticing the effect of diversity-dependent ecological factors (that is, the proximity to diversity saturation). The diversity-to-*K*_eff_ ratio falls below 0.25 in most of the ocean and throughout the Phanerozoic (Fig. 3a–l and Supplementary Video 4), supporting the idea that the dynamics of regional diversity have been systematically operating below *K*_eff_ and, therefore, far from saturation.

Finally, we calculated the diversity-to-*K*_eff_ ratio along the flooded continental regions using the combinations of *K*_min_ and *K*_max_ that resulted from simulations with different parameter values (Extended Data Fig. 4 (insets, grey lines) and Extended Data Table 2), and represent its frequency distributions (Fig. 3m–o). Most of the estimates fall within the exponential growth regime of the logistic function (that is, diversity-to-*K*_eff_ ratio < 0.25). On average, less than 10% of the estimates exceed the threshold of 0.25, and less than only 2% of the estimates, those associated with well-developed diversity hotspots, exceed the threshold of 0.5.

## Discussion

**Extended Data Fig. 7:**
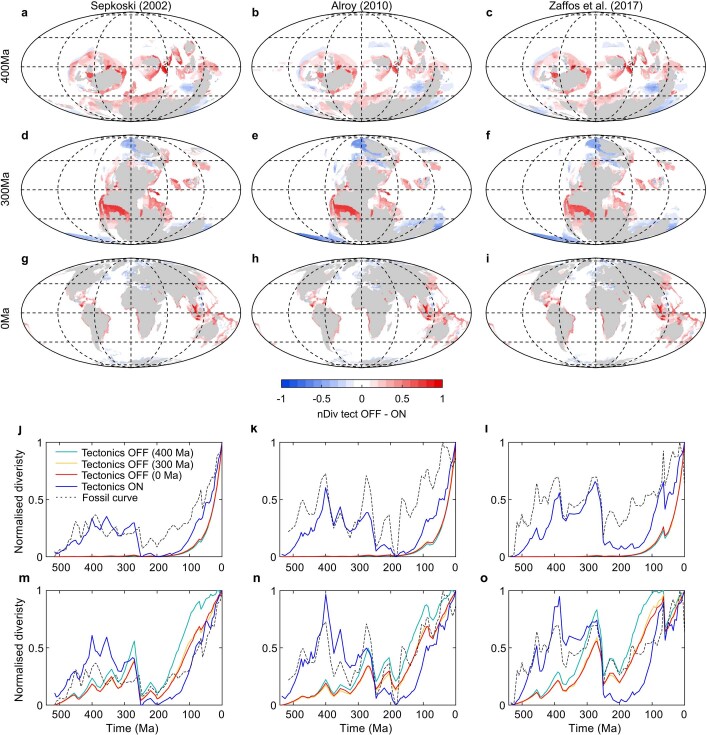
Testing the effect of plate tectonics. **a-i**, Comparison of the results of the calibrated logistic model without plate tectonics and with plate tectonics. The color code represents the difference between the log-transformed normalized diversities (0-1) produced by the model with static palaeogeography (nDiv tectonics OFF) and the model with variable palaeogeography (nDiv tectonics ON) for three time frames (panels row-wise 400 Ma, 300 Ma and 0 Ma) and three mass extinction patterns (panels column-wise Sepkoski^20^, Alroy^21^ and Zaffos et al^22^). **j-l**, Global diversity dynamics produced by the exponential diversification model with static palaeogeography (light blue, yellow and red colour lines correspond to palaeogeographic configurations of 400 Ma, 300 Ma and 0 Ma, respectively) and with variable palaeogeography (blue line) for each of the three mass extinction patterns (panels column-wise Sepkoski^20^, Alroy^21^ and Zaffos et al^22^). The corresponding fossil diversity curve is superimposed on each panel (grey dashed line). **m-o**, As in panels j-l but for the calibrated logistic model.

**Extended Data Fig. 8:**
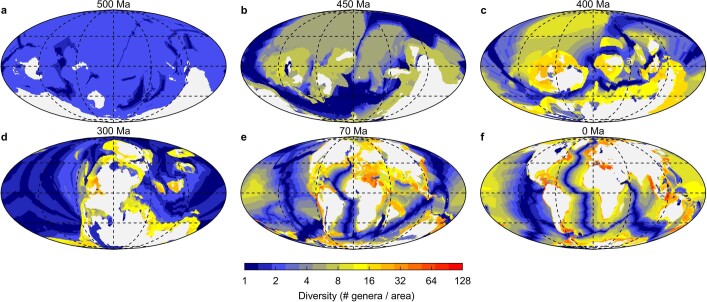
Testing the effect of environmental forcings. Spatial distribution of marine animal diversity (# genera / area) in the Cambrian (Guzhangian, 500 Ma), Late Ordovician (Katian, 450 Ma), Early Devonian (Emsian, 400 Ma), Late Carboniferous (Pennsylvanian, 300 Ma), Late Cretaceous (Maastrichtian, 70 Ma) and present generated by the calibrated logistic model after imposing the pattern of mass extinctions extracted from the fossil diversity curve of Sepkoski^20^. This model run uses the following parameters: *Q*_10_ = 1 (no temperature dependence), *K*_food_ = 0 molC m^−2^y^−1^ (no food dependence), net diversification rate limits (*ρ*_min_ − *ρ*_max_) = 0.001-0.035 Myr^−1^, and a *K*_min_ to *K*_max_ range between 12 and 123 genera.

Our model corroborates earlier claims that Earth’s environmental history^5,25,26^ and the patterns of continental fragmentation and reassembly^22,27,28^ have been major determinants of marine animal diversification. The use of a mechanistic model also enables examination of the probable causes of particular patterns in the fossil record that cannot be deduced from inspection of the fossil diversity curve alone. For example, we found that, in the absence of progressive continental reconfiguration that allows continental shelf habitats to reposition along the latitudinal temperature gradient, diversity grows disproportionately in shelf seas lying permanently within the tropical belt (Methods and Extended Data Fig. 7). We can also test the role of temperature and food dependence of the net diversification rate, and find that, by disabling these relationships in the model, highly unrealistic biogeographical distributions arise, such as the occurrence of diversity hotspots in high latitudes, leaving the growth of diversity solely as a function of habitat age (Extended Data Fig. 8). However, there are also limits to the potential factors that can be accounted for in our modelling. For example, here we do not account for variations through time of seawater oxygenation, owing to uncertainty in both how oxygenation might set limits on maximum diversity as well as in the Phanerozoic history of the atmospheric composition itself^29^. The finite, approximately 5 million year temporal resolution of our time slices also precludes more rapid changes—such as cycles in sea level and continental flooding—from being explicitly accounted for, creating a potential temporal error in the loss or gain of shelf habitat and associated changes in biodiversity.

It has been hypothesized that the Mesozoic marine revolution^12,13^, that is, the emergence of shell-crushing predators and the consequent ecological restructuring of marine ecosystems, was primarily responsible for the increase in global diversity over the last 150 million years. The fact that our model can reproduce such an increase in diversity without the need to invoke evolutionary innovations like the emergence of new modes of predation^12,13^, defence^12,30^, mobility^30^ or reproduction^31^, among others, raises a new hypothesis based on how Earth’s environmental history and palaeogeographical evolution interacted in concert to enable the development of diversity hotspots. We call this the ‘diversity hotspots hypothesis’, which is proposed as a non-mutually exclusive alternative to the hypothesis that evolutionary innovation and new ecospace occupation led this macroevolutionary trend.

With the possible exception of diversity hotspots, our results indicate that the diversity of marine invertebrates has remained below saturation throughout their evolutionary history, shedding light on one of the most controversial topics in evolutionary ecology^1–5,8–11^. A model of taxonomic diversification operating within the exponential growth regime of a logistic function implies that diversity will recover faster than it would if it operated near saturation, with important consequences for post-extinction recovery﻿ dynamics﻿. We envision that our spatially explicit reconstructions of diversity could shed light on other long-standing questions in biogeography and macroevolution, as well as provide a synthetic, spatially resolved history of biodiversity, that can be sampled in different ways to explore sampling biases in the rock record.

## Methods

### Palaeogeographical model

We use palaeogeographical reconstructions describing Earth’s palaeotopography and palaeobathymetry for a series of time slices from 541 Ma to the present day. The reconstructions merge existing models from two published global reconstruction datasets—those of ref. ^32^ and ref. ^33^ (10.5281/zenodo.5348492), which themselves are syntheses of a wealth of previous work.

For continental regions, estimates of palaeoelevation and continental flooding rely on a diverse range of geological evidence, such as sedimentary depositional environments and the spatiotemporal distribution of volcanic activity. For a full description, see a recent review^34^. Together, these data can be used to define the past locations of mountain ranges and palaeoshorelines^34^. For this part of our reconstruction, we used the compilation of ref. ^33^ with updated palaeoshorelines based on depositional environment information in current fossil databases^35^. This compilation comprises 82 palaeotopography maps covering the entire Phanerozoic. Note that each palaeogeographical map is a time slice representing the concatenation of geological data over several million years^36^.

We quantified the impact of using the original compilation of ref. ^33^ on our model results and found only small changes with respect to using the reconstructions with updated palaeoshorelines (Extended Data Fig. 3a–c). Similarly, eustatic sea level is thought to have varied by around 100 m at timescales much shorter than the duration of the time-slices throughout the Phanerozoic^37^, such that the extent of continental flooding could have varied within each time slice by an amount significant for our analysis. For this reason, and to assess the uncertainty of our results to continental palaeogeography in general, we computed additional maps of continental flooding in which the sea level is raised or lowered by 100 m compared with the original palaeo–digital elevation model grids of ref. ^33^ (Extended Data Fig. 3d–f).

For deep-ocean regions, the primary control on seafloor depth is the age of the seafloor, so reconstructing palaeobathymetry relies on constructing maps of seafloor age back in time^38^. As a consequence, we rely on reconstruction models that incorporate a continuous network of plate boundaries. For this part, we used the reconstruction of ref. ^32^ and derived maps of seafloor age from the plate tectonic model using the method of ref. ^39^ for which source code is available at GitHub (https://github.com/siwill22/agegrid-0.1). Palaeobathymetry is derived from the seafloor age maps following the steps outlined in ref. ^38^. It is important to note that seafloor age maps for most of the Phanerozoic (that is, pre-Pangaea times) are not directly constrained by data due to recycling of oceanic crust at subduction zones. Rather, they are model predictions generated by constructing plate motions and plate boundary configurations from the geological and palaeomagnetic record of the continents. Nonetheless, the first-order trends in ocean-basin volume and mean seafloor age are consistent with independent estimates for at least the last 410 million years (Myr)^39^.

The reconstructions of refs. ^32,33^ differ in the precise locations of the continents through time. To resolve this discrepancy, we reverse reconstructed the continental palaeoelevation model of ref. ^33^ to present-day coordinates using their rotation parameters, then reconstructed them back in time using the rotations of ref. ^32^. Owing to the differences in how the continents are divided into different tectonic units, this process leads to some gaps and overlaps in the results^40^, which we resolved primarily through a combination of data interpolation and averaging. Manual adjustments were made to ensure that the flooding history remained consistent with the original palaeotopography in areas in which interpolation gives a noticeably different history of seafloor ages. The resulting palaeotopography maps are therefore defined in palaeomagnetic reference frame^32^ appropriate for use in Earth system models.

For the biodiversity modelling, we generate estimates of the age of the seafloor for discrete points within the oceans and flooded continents, and track these ages through the lifetime of each point (Supplementary Video 5). For the oceans, this is achieved using the method described in ref. ^39^ in which the seafloor is represented by points that are incrementally generated at the mid-ocean ridges for a series of time steps 1 Myr apart, with each point tracked through subsequent time steps based on Euler poles of rotation until either the present-day is reached, or they arrive at a subduction zone and are considered to be destroyed.

For the continents, tracking the location of discrete points is generally simpler as most crust is conserved throughout the timespan of the reconstruction. In contrast to the deep oceans (where we assume that crust is at all times submerged), we model the ‘age’ of the seafloor from the history of continental flooding and emergence within the palaeogeographical interpretation^33^. The continents are seeded with uniformly distributed points at the oldest timeslice (541 Ma) at which they are assigned an age of zero. These points are tracked to subsequent time slices of which the palaeogeography is used to determine whether the point lies within a flooded or emergent region. Points within flooded regions of continents are considered to be seafloor, and the age of this seafloor is accumulated across consecutive time slices where a given point lies within a flooded region. When a point is within an emergent region, the seafloor age is reset to zero. Following this approach, individual points within stable continents may undergo several cycles of seafloor age increasing from zero before being reset. At the continental margins formed during the Pangaea breakup, the age of the seafloor continuously grows from the onset of rifting. Intraoceanic island arcs represent an additional case, which can appear as new tectonic units with the reconstructions at various times. In these cases, we assume that the seafloor has a zero-age at the time at which the intraoceanic arc first develops, then remains predominantly underwater for the rest of its lifetime.

Thus, for each of the 82 palaeogeographical reconstructions, we annotate 0.5° by 0.5° grids as continental, flooded continental shelf or oceanic for later use in model coupling and production of regional diversity maps.

### Palaeoenvironmental conditions under the cGENIE Earth system model

We use cGENIE^41^, an Earth system model of intermediate complexity, to simulate palaeoenvironmental conditions of seawater temperature and organic carbon export production (as a surrogate for food supply) throughout the Phanerozoic (from 541 Ma to the present day).

cGENIE is based on a three-dimensional (3D) ocean circulation model coupled to a 2D energy–moisture balance atmospheric component and a sea-ice module. We configured the model on a 36 × 36 (latitude, longitude) equal area grid with 17 unevenly spaced vertical levels in depth, down to a maximum ocean depth of 5,900 m. The cycling of carbon and associated tracers in the ocean is based on a size-structured plankton ecosystem model with a single (phosphate) nutrient^42,43^, and adopts an Arrhenius-type temperature-dependent scheme for the remineralization of organic matter exported to the ocean interior^44^.

cGENIE provides a spatially resolved representation of ocean physics and biogeochemistry, which is a prerequisite for the present study to be able to reconstruct the spatial patterns of biodiversity in deep time. However, owing to the computational impracticality of generating a single transient simulation of physics (that is, temperature) and biogeochemistry (that is, export production) over the entire Phanerozoic, we therefore generate 30 model equilibria at regular time intervals throughout the Phanerozoic that are subsequently used as inputs for the regional diversification model (see the ‘Model coupling’ section).

We used 30 Phanerozoic palaeogeographical reconstructions through time (~20 Myr evenly spaced time intervals) to represent key time periods. For each continental configuration corresponding to a given age in Earth history, we generate idealized 2D (but zonally averaged) wind speed and wind stress, and 1D zonally averaged albedo forcing fields^45^ required by the cGENIE model using the ‘muffingen’ open-source software (see the ‘Code availability’ section). For each palaeogeographical reconstruction, the climatic forcing (that is, solar irradiance and carbon dioxide concentration) is adapted to match the corresponding geological time interval. The partial pressure of CO_2_ is taken from the recent update of the GEOCARB model^46^. Solar luminosity is calculated using the model of stellar physics of ref. ^47^. We impose modern-day orbital parameters (obliquity, eccentricity and precession). The simulations are initialized with a sea-ice-free ocean, homogeneous oceanic temperature (5 °C) and salinity (34.9‰). As variations in the oceanic concentration of bio-available phosphate remain challenging to reconstruct in the geological past^48,49^, we impose a present-day mean ocean phosphate concentration (2.159 μmol kg^−1^) in our baseline simulations. We quantify the impact of this uncertainty on our model results by conducting additional simulations using half and twice the present-day ocean phosphate concentration (Extended Data Fig. 3g–i). For each ocean phosphate scenario (that is, 0.5×, 1× and 2× the present-day value), each of the 30 model simulations is then run for 20,000 years, a duration ensuring that deep-ocean temperature and geochemistry reach equilibrium. For each model simulation, the results of the mean annual values of the last simulated year are used for the analysis. Note that, although cGENIE makes projections of the distribution of dissolved oxygen ([O_2_]) in the ocean, our diversification model does not currently consider oxygenation to be a limit on diversity. Thus, we assumed a modern atmospheric partial pressure of O_2_ in all 30 palaeo simulations and did not use the resulting projected [O_2_] fields.

### Regional diversification model

We tested two models of diversification—the logistic model and the exponential model—describing the dynamics of regional diversity over time. In both models, the net diversification rate (*ρ*), with units of inverse time (Myr^−1^), varies within a pre-fixed range of values as a function of seawater temperature and food availability. The net diversification rate is then calculated for a given location and time according to the following equation:

1 $$\rho ={\rho }_{\max }-({\rho }_{\max }-{\rho }_{\min })(1-{Q}_{{\rm{temp}}}{Q}_{{\rm{food}}})$$

where *ρ*_min_ and *ρ*_max_ set the lower and upper net diversification rate limits within which *ρ* is allowed to vary, and *Q*_temp_ and *Q*_food_ are non-dimensional limitation terms with values between 0 and 1 that define the dependence of *ρ* on temperature and food, respectively (Extended Data Table 1).

The model considers a direct relationship between seawater temperature, food supply and the rate of net diversification on the basis of the theoretical control that temperature and food supply exert on the rates of origination and extinction (Supplementary Fig. 1). Temperature rise is expected to accelerate the biochemical kinetics of metabolism^50^ and shorten the development times of individuals^51^, leading to higher rates of mutation and origination. Greater food availability increases population sizes, which increases the rates of mutation and reduces the probability of extinction^52^. Furthermore, a large body of observations shows the existence of a positive relationship between resource availability (that is, food supply) and the standing stock of species in marine and terrestrial communities^53,54^. A larger food supply would support a greater number of individuals. A greater diversity of food resources could also lead to a finer partitioning of available resources^55^.

The temperature dependence of *ρ* is calculated using the following equation:

2 $${Q}_{{\rm{temp}}}=\frac{{Q}_{10}^{\frac{T-T\min }{10}}}{{Q}_{10}^{\frac{T\max -T\min }{10}}}$$

where the *Q*_10_ coefficient measures the temperature sensitivity of the origination rate. In equation (2) above, *T* is the seawater temperature (in °C) at a given location and time, and *T*_min_ and *T*_max_ are the 0.01 percentile and the 0.99 percentile, respectively, of the temperature frequency distribution in each time interval. In the model, the values of *T*_min_ and *T*_max_ used to calculate *Q*_temp_ are therefore recomputed for every time interval (~5 Myr) according to the temperature frequency distribution of the corresponding time interval. This enables us to use updated *T*_min_ and *T*_max_ values in each Phanerozoic time interval and to account for the thermal adaptation of organisms to ever changing climate conditions.

The food limitation term is parameterized using a Michaelis–Menten formulation as follows:

3 $${Q}_{{\rm{food}}}=\frac{\text{POC flux}}{\left({K}_{{\rm{food}}}\,+\,\text{POC flux}\right)}$$

where POC flux (mol m^−2^ yr^−1^) is the particulate organic carbon export flux, which is used as a surrogate for food availability, at a given location and time of the simulated seafloor. The parameter *K*_food_ (mol m^−2^ yr^−1^) in equation (3) is the half-saturation constant, that is, the POC flux at which the diversification rate is half its maximum value, provided that other factors were not limiting. These temperature and food supply limitation terms vary in space and time as a result of changes in seawater temperature and particulate organic carbon export rate, respectively, thereby controlling the spatial and temporal variability of *ρ* (Supplementary Video 6).

The net diversification rate becomes negative (1) in the event of mass extinctions or (2) in response to regional-scale processes, such as sea-level fall and/or seafloor deformation along convergent plate boundaries. Mass extinction events are imposed as external perturbations to the diversification model by imputing negative net diversification rates to all active seafloor points (ocean points and flooded continental points) and assuming non-selective extinction. The percentage of diversity loss as well as the starting time and duration of mass extinctions are extracted from three fossil diversity curves of reference^20–22^ (Source Data for Fig. 1). Each of these fossil diversity curves provides different insights into the Phanerozoic history of marine animal diversity based on uncorrected range-through genus richness estimates^20,22^ and sampling standardized estimates^21^. Regional-scale processes—such as sea level fall during marine regressions and/or seafloor destruction at plate boundaries, either by subduction or uplift—are simulated by the combined plate tectonic–palaeoelevation model, and constrain the time that seafloor habitats have to accumulate diversity.

The model assumes non-selective extinction during mass extinction events (that is, the field of bullets model of extinction; everything is equally likely to die, no matter the age of the clade and regardless of adaptation)^56^. However, there is much fossil evidence supporting extinction selectivity^57,58^. It could be argued that higher extinction rates at diversity hotspots would have delayed their subsequent recovery, flattening global diversity trends. This argument is difficult to reconcile with Sepkoski’s genus-level global diversity curve but could be consistent with the standardized diversity curve of ref. ^21^. Similarly, the model is also not suitable for reproducing the explosive radiations of certain taxonomic groups after mass extinctions, which could explain the offset between the model and fossil observations in the early Mesozoic (Fig. 1).

Letting *D* represent regional diversity (number of genera within a given seafloor point) and *t* represent time, the logistic model is formalized by the following differential equation:

4 $$\frac{\partial D\left(t\right)}{\partial t}=\rho D\left[1-\frac{D}{{K}_{{\rm{eff}}}}\right]$$

where *D*(*t*) is the number of genera at time *t* and *K*_eff_ is the effective carrying capacity or maximum number of genera that a given seafloor point (that is, grid cell area after gridding) can carry at that time, *t*. In our logistic model, *K*_eff_ is allowed to vary within a fixed range of values (from *K*_min_ to *K*_max_) as a positive linear function of the POC flux at a given location and time as follows:

5 $${K}_{{\rm{eff}}}={K}_{\max }-\left({K}_{\max }-{K}_{\min }\right)\frac{{\text{POC flux}}_{\max }-\text{POC flux}}{{\text{POC flux}}_{\max }-{\text{POC flux}}_{\min }}$$

where POC flux_min_ and POC flux_max_ correspond to the 0.01 and 0.99 quantiles of the POC flux range in the whole Phanerozoic dataset.

In the logistic model, the net diversification rate decreases as regional diversity approaches its *K*_eff_. The exponential model is a particular case of the logistic model when *K*_eff_ approaches infinity and, therefore, neither the origination rate nor the extinction rate depend on the standing diversities. In this scenario, diversity grows in an unlimited manner over time only truncated by the effect of mass extinctions and/or by the dynamics of the seafloor (creation versus destruction). Thus, the exponential model is as follows:

6 $$\frac{\partial D\left(t\right)}{\partial t}=\rho D$$

where the rate of change of diversity (the time derivative) is proportional to the standing diversity *D* such that the regional diversity will follow an exponential increase in time at a speed controlled by the temperature- and food-dependent net diversification rate. Even if analytical solutions exist for the steady-state equilibrium of the logistic and exponential functions, we solved the ordinary differential equations (4) and (6) using numerical methods with a time lag of 1 Myr to account for the spatially and temporally varying environmental constraints, seafloor dynamics and mass extinction events.

As the analysis of global fossil diversity curves is unable to discern the causes of diversity loss during mass extinctions, our imputation of negative diversification rates could have overestimated the loss of diversity in those cases in which sea level fall, a factor already accounted for by our model, contributed to mass extinction. This effect was particularly recognizable across the Permian–Triassic mass extinction (Extended Data Fig. 6d–f), and supports previous claims that the decline in the global area of the shallow water shelf exacerbated the severity of the end-Permian mass extinction^34^.

### Model coupling

As stated above, the coupled plate tectonic–palaeoelevation (palaeogeographical) model corresponds to a tracer-based model (a Lagrangian-based approach) that simulates and tracks the spatiotemporal dynamics of ocean and flooded continental points. The diversification models start at time 541 Ma with all active points having a *D*_0_ = 1 (one single genus everywhere) and we let points accumulate diversity heterogeneously with time according to seafloor age distributions (for ocean points) and the time that continents have been underwater (for flooded continental points). The ocean points are created at mid-ocean ridges and disappear primarily at subduction zones. Between their origin and demise, the points move following plate tectonic motions and we trace their positions while accumulating diversity. The flooded continental points begin to accumulate diversity from the moment that they are submerged, starting with a *D* value equal to the nearest neighbour flooded continental point with *D* > 1, thereby simulating a process of coastal recolonization (or immigration). The diversification process remains active while the seafloor points remain underwater, but it is interrupted, and *D* set to 0, in those continental points that emerge above sea level. Similarly, seafloor points corresponding to ocean domains disappear in subduction zones, and their diversity is lost. We track the geographical position of the ocean and flooded continental points approximately every 5 Myr, from 541 Ma to the present. Each and every one of the tracked points accumulates diversity over time at a different rate, which is modulated by the environmental history (seawater temperature and food availability) of each point, as described in equations (1)–(3). When a point arrives in an environment with a carrying capacity lower than the diversity it has accumulated through time, we reset the diversity of the point to the value of the carrying capacity, thereby simulating local extinction.

Seawater temperature (*T*) and food availability (POC flux) are provided by the cGENIE model, which has a spatial and temporal resolution coarser than the palaeogeographical model. The cGENIE model provides average seawater *T* and POC flux values in a 36 × 36 equal area grid (grid cell area equivalent to 2° latitude by 10° longitude at the equator) and 30 time slices or snapshots (from 541 Ma to the present: each ~20 Myr time intervals). To have environmental inputs for the 82 time slices of the plate tectonic–palaeoelevation model, we first interpolate the cGENIE original model output data on a 0.5° by 0.5° grid to match the annotated grids provided by the plate tectonic–palaeoelevation model. As the relatively coarse spatial resolution of the cGENIE model prevents rendering the coast–ocean gradients, we assign surface *T* and POC flux at the base of the euphotic zone to the flooded continental shelf grid cells, and deep ocean *T* and POC flux at the bottom of the ocean to the ocean grid cells. As there are time slices without input data of seawater *T* and POC flux, we interpolate/extrapolate seawater *T* and POC flux values into the 0.5° by 0.5° flooded continental shelf and ocean grids independently. Finally, we interpolate values from these 0.5° by 0.5° flooded continental shelf and ocean grids into the exact point locations in each time frame. Thus, each active point is tracked with its associated time-varying *T* and POC flux values throughout its lifetime. On average, 6,000 flooded continental points and 44,000 oceanic points were actively accumulating diversity in each time frame. The model cannot simulate the singularities of relatively small enclosed seas for which the spatial resolution of the palaeogeographical and Earth system models is insufficient to capture relevant features (such as palaeobathymetry, seawater temperature) in detail. The method is also likely to underestimate the diversity of epeiric (inland) seas due to the difficulty of simulating immigration, a process that is strongly influenced by the effect of surface ocean currents and is not considered here. However, as stated above, the model considers recolonization of recently submerged areas by marine biota from nearby coastal environments, which partially explains coastal immigration.

### Estimation of global diversity from regional diversity

Our regional diversity maps are generated by separately interpolating ocean point diversity and flooded continental point diversity into the 0.5° by 0.5° annotated grids provided by the palaeogeographical model. We calculate global diversity at each time step from each of the regional diversity maps following a series of steps to integrate diversity along line transects from diversity peaks (maxima) to diversity troughs (minima) (Extended Data Fig. 1). To select the transects, first, we identify on each of the regional diversity maps the geographical position of the diversity peaks. We identify local maxima (that is, grid cells with diversity greater than their neighbour cells), and define the peaks as those local maxima with diversity greater than the 0.75 quantile of diversity values in all local maxima in the map. In the case of grid cells with equal neighbour diversity, the peak is assigned to the grid cell in the middle. We subsequently identify the geographical position of the diversity troughs, which are defined as newly formed ocean grid cells (age = 0 Myr) and, therefore, with diversities equal to one. The troughs are mostly located at mid-ocean ridges.

On each of the 82 spatial diversity maps, we trace a line transect from each diversity peak to its closest trough, provided that the transect does not cross land in more than 20% of the grid cells along the linear path (Supplementary Video 7). On average, for each spatial diversity map, we trace 400 (*σ* = ±75) linear transects. This sampling design gives rise to transects of different lengths, which may bias the estimates of global diversity. To minimize this bias, we cut the tail of the transects to have a length of 555 km equivalent to 5° at the equator. We tested an alternative cut-off threshold, 1,110 km, and the results do not alter the study’s conclusions.

We apply Bresenham’s line algorithm^59^ to detect the grid cells crossed by the transects and annotate their diversity. To integrate regional diversity along the transects, we developed a method to simplify the scenario of peaks and troughs heterogeneously distributed on the 2D diversity maps. The method requires (1) a vector (the transect) of genus richness (**α**_*n*_) at *n* different locations (grids) arranged in a line (1D) of *L* grids, and (2) a coefficient of similarity (*V*_*n*,*n* + 1_) between each two neighbouring locations, *n* and *n* + 1. *V*_*n*,*n* + 1_, the coefficient of similarity, follows a decreasing exponential function with distance between locations. The number of shared genera between *n* and *n* + 1 is *V*_*n*,*n* + 1_ × min(**α**_n_; **α**_*n* + 1_). We integrate diversity from peaks to troughs and assume that, along the transect, **α**_*n* + 1_ is lower than **α**_*n*_. We further assume that the genera present in *n* and *n* + 2 cannot be absent from *n* + 1. Using this method, we integrate the transect’s diversity (*γ*_*i*_) using the following equation:

7 $${\gamma }_{i}={ {\mathbf{\upalpha}}}_{1}+{\sum }_{n=1}^{L-1}\left(1-{V}_{n,n+1}\right){ {\mathbf{\upalpha}}}_{n+1}$$

To integrate the diversity of all transects (*γ*_*i*_) on each 2D diversity map (or time slice), we apply the same procedure as described above (Extended Data Fig. 1). We first sort the transects in descending order from the highest to the lowest diversity. We then assume that the number of shared genera between transect *i* and the rest of the transects with greater diversity {1, 2, …, *i* − 1} is given by the distance of its peak to the nearest neighbour peak (NN(*i*)) of those already integrated {1, 2, …, *i* − 1}. Thus, we perform a zigzag integration of transects’ diversities down gradient, from the greatest to the poorest, weighted by the nearest neighbour distance among the peaks already integrated. As a result, the contribution of each transect to global diversity will depend on its diversity and its distance to the closest transect out of all those transects already integrated. Using this method, we linearize the problem to simplify the cumbersome procedure of passing from a 2D regional diversity map to a global diversity estimate without knowing the identity (taxonomic affiliation) of the genera. If *γ*_total_ is the global diversity at time *t*:

8 $${\gamma }_{{\rm{total}}}={\gamma }_{1}+{\sum }_{i=2}^{j}\left(1-{V}_{{\rm{NN}}\left(i\right),i}\right){\gamma }_{i}$$

Finally, the resulting global estimates are plotted against the midpoint value of the corresponding time interval to generate a synthetic global diversity curve. To compare the global diversity curves produced by the diversification models with those composed from the fossil record, Lin’s CCC^60^ is applied to the data normalized to the min–max values of each time series (that is, rescaled within the range 0–1). Lin’s CCC combines measures of both precision and accuracy to determine how far the observed data deviate from the line of perfect concordance (that is, the 1:1 line). Lin’s CCC increases in value as a function of the nearness of the data’s reduced major axis to the line of perfect concordance (the accuracy of the data) and of the tightness of the data around its reduced major axis (the precision of the data).

The time series of global diversity generated from the fossil record and from the diversification model exhibit serial correlation and the resulting CCCs are therefore inflated. The use of methods for analysing non-zero autocorrelation time series data, such as first differencing or generalised least squares regression, enables high-frequency variations along the time series to be taken into account. However, the relative simplicity of our model, which was designed to reproduce the main Phanerozoic trends in global diversity, coupled with the fact that biases in the fossil data would introduce uncertainty into the analysis, leads us to focus our analysis on the long-term trends, obviating the effect of autocorrelation.

### Model parameterization and calibration

The diversification models are parameterized assuming a range of values that constrain the lower and upper limits of the genus-level net diversification rate (*ρ*_min_ and *ρ*_max_, respectively) (Extended Data Table 1) according to previously reported estimates from fossil records (figures 8 and 11 of ref. ^5^). A range of realistic values is assigned for the parameters *Q*_10_ and *K*_food_, determining, respectively, the thermal sensitivity and food dependence of the net diversification rate. We test a total of 40 different parameter combinations (Extended Data Table 2). The resulting estimates of diversity are then compared against the fossil diversity curves of ref. ^20^, ref. ^21^ or ref. ^22^, and the 15 parameter combinations providing the highest CCCs are selected.

The results of the logistic diversification model rely on the values of the minimum and maximum carrying capacities (*K*_min_ and *K*_max_, respectively) within which the spatially resolved effective carrying capacities (*K*_eff_) are allowed to vary. The values of *K*_min_ and *K*_max_ are therefore calibrated by running 28 simulations of pair-wise *K*_min_ and *K*_max_ combinations increasing in a geometric sequence of base 2, from 2 to 256 genera (Extended Data Fig. 4). We perform these simulations independently for each of the 15 parameter settings selected previously (Extended Data Fig. 4 and Extended Data Table 2). Each combination of *K*_min_ and *K*_max_ produces a global diversity curve, which is evaluated as described above using Lin’s CCC.

Calculating estimates of global diversity from regional diversity maps in the absence of information on genus-level taxonomic identities requires that we assume a spatial turnover of taxa with geographical distance (Extended Data Fig. 1). Distance-decay curves are routinely fitted by calculating the ecological similarity (for example, the Jaccard similarity index) between each pair of sampling sites, and fitting an exponential decay function to the points on a scatter plot of similarity (*y* axis) versus distance (*x* axis). Following this method, we fit an exponential decay function to the distance–decay curves reported in ref. ^61^, depicting the decrease in the Jaccard similarity index (*J*) of fossil genera with geographical distance (great circle distance) at different Phanerozoic time intervals:

9 $$J={J}_{{\rm{o}}{\rm{f}}{\rm{f}}}+(\,{J}_{max}\text{-}{J}_{{\rm{o}}{\rm{f}}{\rm{f}}}){{\rm{e}}}^{-\lambda \times {\rm{d}}{\rm{i}}{\rm{s}}{\rm{t}}{\rm{a}}{\rm{n}}{\rm{c}}{\rm{e}}}$$

where *J*_off_ = 0.06 (n.d.) is a small offset, *J*_max_ = 1.0 (n.d.) is the maximum value of the genus-based Jaccard similarity index and λ = 0.0024 (km^−1^) is the distance-decay rate.

The Jaccard similarity index (*J*) between consecutive points *n* and *n* + 1 is bounded between 0 and min(**α**_*n*_; **α**_*n* + 1_)/max(**α**_*n*_; α_*n* + 1_). A larger value for *J* would mean that there are more shared genera between the two communities than there are genera within the least diverse community, which is ecologically absurd. However, using a single similarity decay function can lead the computed value of *J* to be locally larger than min(**α**_*n*_; **α**_*n* + 1_)/max(**α**_*n*_; **α**_*n* + 1_). To prevent this artefact, we use the Simpson similarity index or ‘overlap coefficient’ (*V*) instead of *J*. *V* corresponds to the percentage of shared genera with respect to the least diverse community (min(**α**_*n*_ ; **α**_*n* + 1_)). *V* is bounded between 0 and 1, whatever the ratio of diversities. As the pre-existing estimates of similarity are expressed using *J* (ref. ^61^), we perform the conversion from *J* to *V* using the algebraic expression *V* = (1 + *R*) × *J*/(1 + *J*) where *R* = max(**α**_*n*_; **α**_*n* + 1_)/min(**α**_*n*_; **α**_*n* + 1_) (Supplementary Note 1). In the cases in which *J* exceeds the min(**α**_*n*_; **α**_*n* + 1_)/max(**α**_*n*_; **α**_*n* + 1_), *V* becomes >1 and, in those cases, we force *V* to be <1 by assuming *R* = 1, that is **α**_n_ = **α**_*n* + 1_.

The model considers a single distance-decay function for the spatial turnover of taxonomic composition. However, the degree of provinciality (that is, the partitioning of life into distinct biogeographical units) varies in space and time as a result of environmental gradients^62^ and plate tectonics^63^. In fact, the increase in provinciality has been invoked as the main driver of the increase in global diversity, especially in the Late Cretaceous and Cenozoic^22,62,63^. This is a deficiency of the model. Unfortunately, information on the extent to which marine provinciality has varied in space and time throughout the Phanerozoic is limited^61,62^, and there is no simple (mechanistic) way to implement different distance-decay functions of taxonomic similarity in the model. There is a clear difference between longitudinal and latitudinal distance, the latter being a more significant source of taxonomic turnover^62^. This effect would add to the observation that tropical diversity hotspots became more prominent towards the end of the Phanerozoic, offering two complementary explanations for the increase in diversity in the Mesozoic: (1) favourable conditions for the development of diversity hotspots and (2) a higher degree of provinciality.

### Fossil data

We used three fossil diversity curves of reference^20–22^ to (1) extract the patterns of mass extinctions (starting time, duration and magnitude) imposed on the model, and (2) compare the global diversity curves produced by the model with those generated from fossil data. Sepkoski’s global diversity curve corresponds to marine invertebrates listed in Sepkoski’s published marine genus compendium^20^ (data downloaded from Sepkoski’s Online Genus Database; http://strata.geology.wisc.edu/jack/). The global diversity curves of refs. ^21,22^ are digitized from the original sources, that is, figure 3 of ref. ^21^ and figure 2a of ref. ^22^, respectively, using the free software XYscan. The curve reported by ref. ^21^ corresponds to genus-richness estimates obtained after correcting for sampling effort using the shareholder quorum subsampling technique. This curve is binned at approximately 11 Myr time intervals and includes non-tetrapod marine animals of which Anthozoa, Trilobita, Ostracoda, Linguliformea, Articulata, Bryozoa, Crinoidea, Echinoidea, Graptolithina, Conodonta, Chondrichthyes, Cephalopoda, Gastropoda and Bivalvia are the major taxonomic groups. The curve reported in ref. ^22^ corresponds to 1 Myr range-through richness estimates of marine skeletonized invertebrate genera including Brachiopoda, Bivalvia, Anthozoa, Trilobita, Gastropoda, Crinoidea, Blastoidea, Edrioasteroidea, Ammonoidea, Nautiloidea and Bryozoa. All digitized (and interpolated) diversity data and the net diversification rate data imputed by the model to simulate mass extinctions are provided as Source Data for Fig. 1.

The choice to analyse the data for all animals is somewhat in conflict with the fact that, in the open ocean, photosynthetic primary production and the flux of organic matter at the bottom of the water column are decoupled. This decoupling leads to contrasting differences in the amount of food available to the planktonic/nektonic and benthic communities yet, in the model, we assume that the amount of organic carbon reaching the seafloor is a proxy for food supply. Given that most of the diversity is concentrated in shallow shelf seas, this assumption is likely to be of only relatively minor importance in a global context.

### OBIS data

We use the occurrence records of genera belonging to two of the most diverse marine invertebrate groups: subphylum Crustacea and phylum Mollusca, as downloaded from the Ocean Biodiversity Information System (OBIS) on 22 October 2021 (www.obis.org). The list of genera is validated with the genera names in WoRMS (https://www.marinespecies.org) and only the accepted, extant and marine names were selected for the analysis. This corresponds to a total of 10,018,142 records of 9,750 genera (6,540,489 records and 5,533 genera of crustaceans and 3,477,653 records and 4,217 genera of molluscs) collected from 1920 to 2021. The records are gridded into hexagons (800,000 km^2^ at the equator) to account for different gamma (regional) diversity across latitudes, otherwise, a bias would occur in the resulting estimates. To ensure sufficient sampling size, we selected only those hexagons with more than or equal to 10 occurrence records and with more than three genera. Furthermore, we use the frequencies of the genera to estimate the number of unobserved genera per hexagon. We do so by extrapolating the number of genera on the basis of a bias-corrected Chao estimate according to the tail of rare genera (that is, those genera that have only one or two occurrence records in a hexagon)^64,65^. The final number of genera per hexagon is the sum of the observed and unobserved estimates of genera. The analysis is performed with the package vegan^66^ in R v.4.1.2. Finally, we spatially overlap the hexagons and 0.5° × 0.5° square grid to match the map of the palaeo analysis and extract the value of the diversity index per coastal grid in QGIS v.3.22.0. The comparison between model and observations is made on the normalized diversities (0–1) bounded between the 0.05 and 0.95 quantiles to minimize the effect of outliers in the observed pattern. These diversity data are provided as Source Data for Fig. 2.

### Testing a static (null) palaeogeographical model

To evaluate the effect of palaeogeography on global diversity dynamics, we carry out simulations for three static palaeogeographical configurations: the Devonian (400 Ma), the Carboniferous (300 Ma) and the present. For each of these three configurations, the model runs for 541 Myr in a ‘static mode’, that is, diversity accumulates steadily at a pace determined by the temperature and food assigned to each grid at the selected static configuration. Mass extinctions are imposed the same way we do in the default model with variable palaeogeography. The test is performed for the exponential diversification model and the calibrated logistic model and for each of the three mass extinction patterns^20–22^. Extended Data Fig. 7a–i shows the differences between the log-transformed normalized diversities (between 0 and 1) produced by the calibrated logistic model with static palaeogeography (nDiv tectonics OFF) and with variable palaeogeography (nDiv tectonics ON). Red and blue colours denote, respectively, the extent to which the static model produces diversity estimates above or below those produced by the model with plate tectonics. Tropical regions are dominated by reddish colours indicating that the static model particularly overestimates diversity in these regions, where high temperatures accelerate diversification. In the absence of plate tectonics, the model leads to a scenario of uncontrolled diversity growth (mainly in the tropical shelf seas; reddish areas on maps) such that even mass extinctions cannot dampen diversity growth (Extended Data Fig. 7j–l) and only effective carrying capacities prevent diversity from running away (Extended Data Fig. 7m–o). A static geographical configuration also prevents diversity hotspots from disappearing at convergent plate boundaries. These results support the idea that Earth’s palaeogeographical evolution and sea level changes, by creating, positioning and destroying seafloor habitats, have had a key role in constraining the growth of diversity throughout the Phanerozoic.

### Reporting summary

Further information on research design is available in the Nature Research Reporting Summary linked to this paper.

## Online content

Any methods, additional references, Nature Research reporting summaries, source data, extended data, supplementary information, acknowledgements, peer review information; details of author contributions and competing interests; and statements of data and code availability are available at 10.1038/s41586-022-04932-6.

### Supplementary information

Supplementary Information Supplementary Fig. 1 and Supplementary Note 1, and descriptions for Supplementary Videos 1–7.

Reporting Summary

Peer Review File

Supplementary Video 1 The saturated logistic model. Full Phanerozoic sequences of the spatial distribution maps of diversity generated by the saturated logistic model after imposing the mass extinction pattern extracted from the fossil diversity curves of refs. ^20^^,21,^^22^, respectively.

Supplementary Video 2 The exponential model. Full Phanerozoic sequences of the spatial distribution maps of diversity generated by the exponential model after imposing the mass extinction pattern extracted from the fossil diversity curves of refs. ^20,2^^1,^^22^, respectively.

Supplementary Video 3 The calibrated logistic model. Full Phanerozoic sequences of the spatial distribution maps of diversity generated by the calibrated logistic model after imposing the mass extinction pattern extracted from the fossil diversity curves of refs. ^20,^^21,^^22^, respectively.

Supplementary Video 4 The diversity-to-carrying capacity ratio. Full Phanerozoic sequences of the spatial distribution maps of the diversity-to-carrying capacity (*K*_eff_) ratio generated by the calibrated logistic model after imposing the mass extinction pattern extracted from the fossil diversity curves of refs. ^20,^^21,^^22^, respectively.

Supplementary Video 5 Time-for-speciation. Full Phanerozoic sequence of the spatial distributions of time-for-speciation.

Supplementary Video 6 Spatially resolved net diversification rate. Full Phanerozoic sequences of the spatial distributions maps of net diversification rate using as model parameters, *Q*_10_ = 1.75, *K*_food_ = 0.5 molC m^−2^ yr^−^^1^ and net diversification rate limits (*ρ*_min_ − *ρ*_max_) = 0.001-0.035 Myr^−1^. The patterns of mass extinctions extracted from the fossil diversity curves of refs. ^20,^^21,^^22^ are represented as zero net diversification rate across the ocean (that is, the entire ocean turns blue).

Supplementary Video 7 Model diversity sampling. An example of how line transects are drawn from diversity peaks to diversity troughs during the model diversity sampling process.

### Source data

Source Data Fig. 1

Source Data Fig. 2

## Figures and Tables

**Extended Data Table 1:**
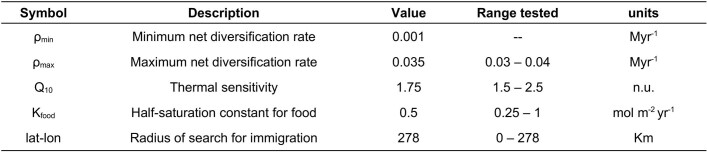
Model parameters and range of parameter values tested

**Extended Data Table 2:**
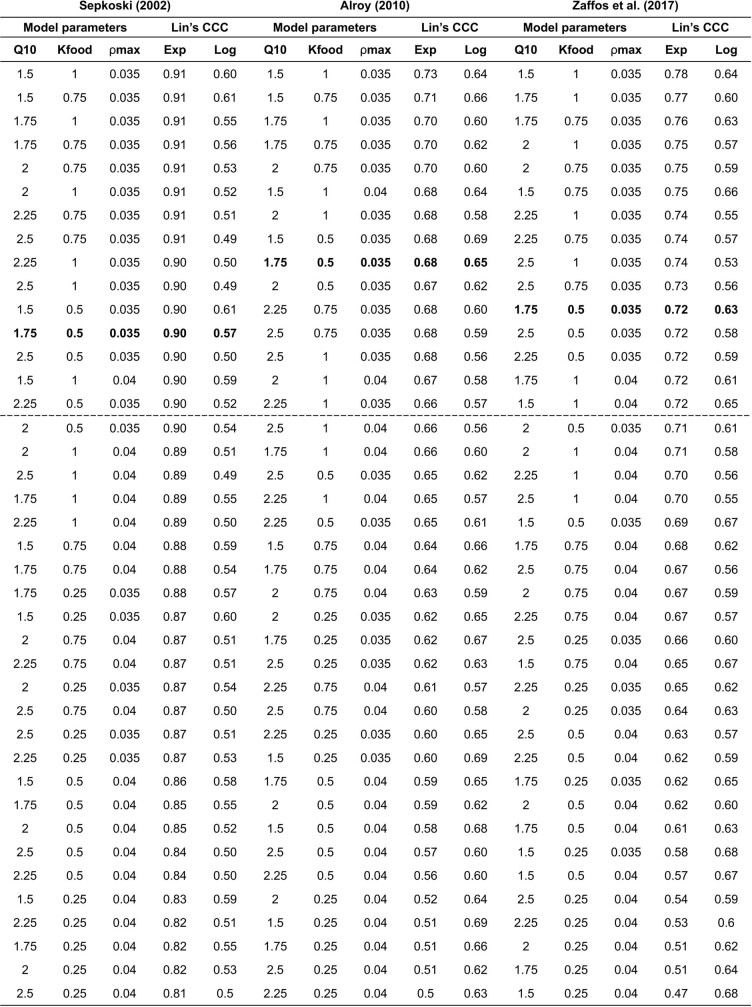
Lin’s Concordance Correlation Coefficient (CCC) using different parameter settings in the model

The CCCs are for the relationship between the normalized diversities estimated from the fossil record and those generated by the exponential (Exp) and the logistic (Log) models. The 15 combinations of model parameters that gave the highest CCC for each mass extinction pattern were selected. Of these, the combination that gave the highest CCC for the relationship between the fossil diversities and the diversities generated by the calibrated logistic model was selected as the best (marked in bold) and used by default to run the model.

## Data Availability

Fossil diversity digitized data and those data downloaded from the Sepkoski’s Online Genus Database (http://strata.geology.wisc.edu/jack/) are provided as source data with this paper. Diversity data downloaded from the Ocean Biodiversity Information System (OBIS) database (www.obis.org) are also provided as source data with this paper. Source data are provided with this paper.

## Code availability

The coupled palaeogeographical–diversification model presented here uses input data of seafloor age distributions and palaeoenvironmental conditions from the siwill22/agegrid-0.1 v1-alpha palaeogeographical model and the cGENIE Earth system model, respectively. The code used to reconstruct seafloor age distributions from GPlates full-plate tectonic reconstructions is available at Zenodo (10.5281/zenodo.3271360). The code for the version of the ‘muffin’ release of the cGENIE Earth system model used in this paper is tagged as v0.9.30, and is available at Zenodo (10.5281/zenodo.6676435). Configuration files for the specific experiments presented in the paper can be found in the directory: genie-userconfigs/PUBS/published/Cermeno_et_al.2022. Details of the experiments, plus the command line needed to run each one, are given in the readme.txt file in that directory. The code for the muffingen boundary file generator, is available at Zenodo (10.5281/zenodo.6676451). The code for the coupled palaeogeographical–diversification model is available at Zenodo (10.5281/zenodo.6535496). The code is also available at GitHub (https://github.com/CarmenGarciaComas/INDITEK); this is the recommended mode of access as it will contain any updates and clarifications.
