# Supplementary material for: Post-extinction recovery of the Phanerozoic oceans and biodiversity hotspots
Source: Nature. 2022 Jul 13;607(7919):507–11. doi: 10.1038/s41586-022-04932-6 (PMC9300466; doi:10.1038/s41586-022-04932-6)
Supplement: Supplementary file 1 — Supplementary Fig. 1 and Supplementary Note 1, and descriptions for Supplementary Videos 1–7. [file 41586_2022_4932_MOESM1_ESM.pdf]

nature portfolio

https://doi.org/10.1038/s41586-022-04932-6

# Supplementary information

# Post-extinction recovery of the Phanerozoic oceans and biodiversity hotspots

In the format provided by the authors and unedited

Supplementary Information

# Post-extinction recovery of the Phanerozoic oceans and biodiversity hotspots

Pedro Cermeño[#,1,*], Carmen García-Comas[#,1,*], Alexandre Pohl[2,3], Simon Williams[4,7], Michael J. Benton[5], Chhaya Chaudhary[6], Guillaume Le Gland[1], R. Dietmar Müller[7], Andy Ridgwell[2], Sergio M. Vallina[8]

[1]Institut de Ciències del Mar, Consejo Superior de Investigaciones Científicas, Barcelona, Spain.
[2]Department of Earth and Planetary Sciences, University of California, Riverside, Riverside, CA, USA.
[3]Biogéosciences, UMR 6282, UBFC/CNRS, Université Bourgogne Franche-Comté, Dijon, France.
[4]State Key Laboratory of Continental Dynamics, Department of Geology, Northwest University, Xi'an, China.
[5]School of Earth Sciences, University of Bristol, Bristol, UK.
[6]Alfred Wegener Institute, Helmholtz Centre for Polar and Marine Research, Bremerhaven, Germany.
[7]EarthByte Group, School of Geosciences, University of Sydney, Sydney, Australia.
[8]Instituto Español de Oceanografía, Consejo Superior de Investigaciones Científicas, Gijón, Spain.

[#]Contributed equally

***To whom correspondence should be addressed:**
*pedrocermeno@icm.csic.es*
*cgcomas@icm.csic.es*

**This Supplementary Information includes:**

Supplementary Figure 1

Captions for Supplementary Videos 1 to 7

Supplementary Note 1

**Further Supplementary Materials:**

Supplementary Videos 1 to 7. Video format: *.mp4 (H.264 encoding)

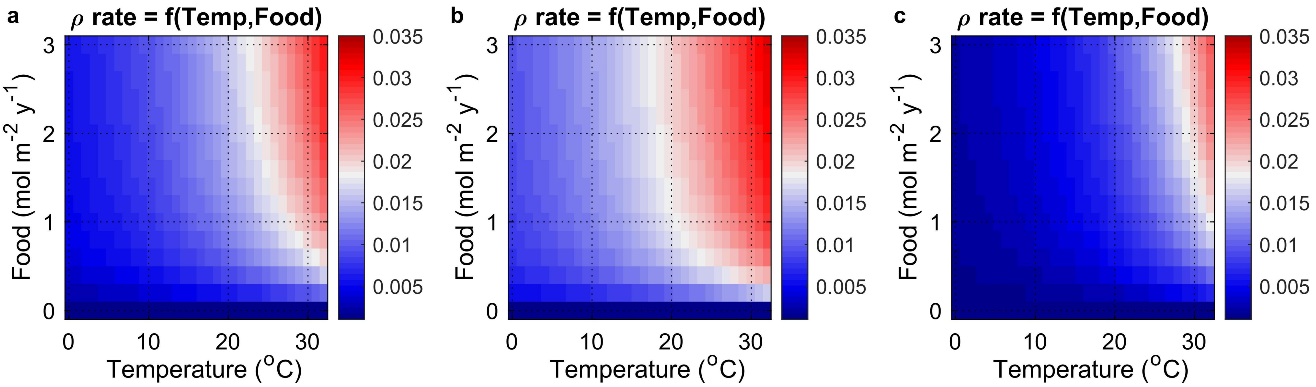


**Supplementary Figure 1: Interactive effect of seawater temperature and food supply on net diversification rate. a**, Combined effect of seawater temperature and food supply on net diversification rate (ρ) for the set of parameters used to run the main simulations (i.e. $Q_{10}$ = 1.75; $K_{food}$ = 0.5 mol C $m^{-2}$ $y^{-1}$; ρ = 0.001 - 0.035 $Myr^{-1}$). **b**, Same as a but for two extreme parameter settings ($Q_{10}$ = 1.5; $K_{food}$ = 0.25 mol C $m^{-2}$ $y^{-1}$; ρ = 0.001 - 0.035 $Myr^{-1}$, and **c**, $Q_{10}$ = 2.5; $K_{food}$ = 1 mol C $m^{-2}$ $y^{-1}$; ρ = 0.001 - 0.035 $Myr^{-1}$).

**Captions for Supplementary Videos 1 to 7.**

**Supplementary Video 1. The saturated logistic model.** Full Phanerozoic sequences of the spatial distribution maps of diversity generated by the saturated logistic model after imposing the mass extinction pattern extracted from the fossil diversity curves of Sepkoski[20], Alroy[21] and Zaffos et al[22], respectively. Video format: *.mp4 (H.264 encoding).

**Supplementary Video 2. The exponential model.** Full Phanerozoic sequences of the spatial distribution maps of diversity generated by the exponential model after imposing the mass extinction pattern extracted from the fossil diversity curves of Sepkoski[20], Alroy[21] and Zaffos et al[22], respectively. Video format: *.mp4 (H.264 encoding).

**Supplementary Video 3. The calibrated logistic model.** Full Phanerozoic sequences of the spatial distribution maps of diversity generated by the calibrated logistic model after imposing the mass extinction pattern extracted from the fossil diversity curves of Sepkoski[20], Alroy[21] and Zaffos et al[22], respectively. Video format: *.mp4 (H.264 encoding).

**Supplementary Video 4. The diversity-to-carrying capacity ratio.** Full Phanerozoic sequences of the spatial distribution maps of the diversity-to-carrying capacity ($K_{eff}$) ratio generated by the calibrated logistic model after imposing the mass extinction pattern extracted from the fossil diversity curves of Sepkoski[20], Alroy[21] and Zaffos et al[22], respectively. Video format: *.mp4 (H.264 encoding).

**Supplementary Video 5. Time-for-speciation.** Full Phanerozoic sequence of the spatial distributions of time-for-speciation. Video format: *.mp4 (H.264 encoding).

**Supplementary Video 6. Spatially-resolved net diversification rate**. Full Phanerozoic sequences of the spatial distributions maps of net diversification rate using as model parameters, $Q_{10}$ = 1.75, $K_{food}$ = 0.5 molC $m^{-2}y^{-1}$ and net diversification rate limits ($\rho_{min}$ - $\rho_{max}$) = 0.001-0.035 $Myr^{-1}$. The patterns of mass extinctions extracted from the fossil diversity curves of Sepkoski[20], Alroy[21] and Zaffos et al[22] are represented as zero net diversification rate across the ocean (i.e., the entire ocean turns blue). Video format: *.mp4 (H.264 encoding).

**Supplementary Video 7. Model diversity sampling.** An example of how line transects are drawn from diversity peaks to diversity troughs during the model diversity sampling process. Video format: *.mp4 (H.264 encoding).

**Supplementary Note 1. Converting Jaccard coefficient to Overlap coefficient.**

The Jaccard similarity index (J) is the metric most commonly used to express the similarity between two communities. Let us call the intersection of two samples $A_n \cap A_{n+1}$ and their union $A_n \cup A_{n+1}$. The cardinal (number of elements) of a set will be represented by vertical bars, i.e. $\alpha_n = |A_n|$. The Jaccard similarity (J) of $A_n$ and $A_{n+1}$ is then defined as the cardinal of the intersection divided by that of the union:

$$J(A_n, A_{n+1}) = \frac{|A_n \cap A_{n+1}|}{|A_n \cup A_{n+1}|} = \frac{|A_n \cap A_{n+1}|}{|A_n| + |A_{n+1}| - |A_n \cap A_{n+1}|}$$

The J index between points n and n+1 is bounded between 0 and $min(\alpha_n; \alpha_{n+1})/max(\alpha_n; \alpha_{n+1})$, where $\alpha_n$; $\alpha_{n+1}$ are the diversities of two samples. A larger value for J (J > 1) would mean that there are more shared species between the two communities than there are species within the least diverse community, which is ecologically absurd. Yet, using a single similarity decay function can lead the computed value of J to be locally larger than $min(\alpha_n; \alpha_{n+1})/max(\alpha_n; \alpha_{n+1})$. To correct this artifact, we used the overlap coefficient (V) instead of J. The overlap coefficient is bounded between 0 and 1, whatever the ratio of diversities. Therefore, using an overlap decay function never creates artifacts.

The overlap coefficient (V), also known as the Szymkiewicz–Simpson coefficient, is defined as the cardinal of the intersection divided by that of the smallest set:

$$V(A_n, A_{n+1}) = \frac{|A_n \cap A_{n+1}|}{(min(|A_n|, |A_n + 1|))}$$

Without loss of generality, let us consider that $\alpha_{n+1}$ is smaller than $\alpha_n$. We will call $R = \alpha_n/\alpha_{n+1}$ the ratio of the two cardinals. V can be estimated from J and vice-versa as follows:

$$V(A_n, A_{n+1}) = J(A_n, A_{n+1}) \frac{|A_n| + |A_{n+1}| - |A_n \cap A_{n+1}|}{|A_{n+1}|} = J(A_n, A_{n+1})(1 + R - V(A_n, A_{n+1}))$$

$$J(A_n, A_{n+1}) = \frac{V(A_n, A_{n+1})}{1 + R - V(A_n, A_{n+1})}$$

$$V(A_n, A_{n+1}) = J(A_n, A_{n+1})(1 + R) - J(A_n, A_{n+1}) V(A_n, A_{n+1})$$

$$V(A_n, A_{n+1})(1 + J(A_n, A_{n+1})) = J(A_n, A_{n+1})(1 + R)$$

$$V(A_n, A_{n+1}) = \frac{(1 + R) J(A_n, A_{n+1})}{1 + J(A_n, A_{n+1})}$$

$$V = \frac{\left[1 + \frac{max(\alpha_n, \alpha_{n+1})}{min(\alpha_n, \alpha_{n+1})}\right] J}{1 + J}$$
