# Supplementary material for: Post-extinction recovery of the Phanerozoic oceans and biodiversity hotspots
Source: Nature. 2022 Jul 13;607(7919):507–11. doi: 10.1038/s41586-022-04932-6 (PMC9300466; doi:10.1038/s41586-022-04932-6)
Supplement: Supplementary file 2 — Reporting Summary [file 41586_2022_4932_MOESM2_ESM.pdf]

nature portfolio

Corresponding author(s): Pedro Cermeño

Last updated by author(s): May 16, 2022

Reporting Summary

Nature Portfolio wishes to improve the reproducibility of the work that we publish. This form provides structure for consistency and transparency in reporting. For further information on Nature Portfolio policies, see our Editorial Policies and the Editorial Policy Checklist.

Statistics

For all statistical analyses, confirm that the following items are present in the figure legend, table legend, main text, or Methods section.

n/a	Confirmed	
☐	☒	The exact sample size (*n*) for each experimental group/condition, given as a discrete number and unit of measurement
☐	☒	A statement on whether measurements were taken from distinct samples or whether the same sample was measured repeatedly
☒	☐	The statistical test(s) used AND whether they are one- or two-sided<br>*Only common tests should be described solely by name; describe more complex techniques in the Methods section.*
☒	☐	A description of all covariates tested
☐	☒	A description of any assumptions or corrections, such as tests of normality and adjustment for multiple comparisons
☐	☒	A full description of the statistical parameters including central tendency (e.g. means) or other basic estimates (e.g. regression coefficient) AND variation (e.g. standard deviation) or associated estimates of uncertainty (e.g. confidence intervals)
☒	☐	For null hypothesis testing, the test statistic (e.g. *F*, *t*, *r*) with confidence intervals, effect sizes, degrees of freedom and *P* value noted<br>*Give P values as exact values whenever suitable.*
☒	☐	For Bayesian analysis, information on the choice of priors and Markov chain Monte Carlo settings
☒	☐	For hierarchical and complex designs, identification of the appropriate level for tests and full reporting of outcomes
☒	☐	Estimates of effect sizes (e.g. Cohen's *d*, Pearson's *r*), indicating how they were calculated

Our web collection on statistics for biologists contains articles on many of the points above.

Software and code

Policy information about availability of computer code

Data collection	The coupled paleogeographic-diversification model presented here uses input data of seafloor age distributions and paleoenvironmental conditions from the siwill22/agegrid-0.1 v1-alpha paleogeographic model (DOI: 10.5281/zenodo.3271360) and the cGENIE Earth System Model (DOI: 10.5281/zenodo.4618023), respectively. Additionally, all data inputs required to run the coupled paleogeographic-diversification model are available along with the model code on GitHub (https://github.com/CarmenGarciaComas/INDITEK).
Data analysis	The model is written in MATLAB 2013b and tested with MATLAB 2021a in a MacOS 2.3 GHz 8-Core Intel Core i9, and with MATLAB 2020b on Windows with a 2.5 GHz Intel i5-3210M and on Linux Debian with a 2.6 GHz Intel Core 9th Gen i9-9980HK processor. The code for the coupled palaeogeographic-diversification model is assigned a DOI: 10.5281/zenodo.6535496. The code used to reconstruct seafloor age distributions from GPlates full-plate tectonic reconstructions is assigned a DOI: 10.5281/zenodo.3271360. The code for the version of the 'muffin' release of the cGENIE Earth System Model used in this study, is tagged as v0.9.20, and is assigned a DOI: 10.5281/zenodo.4618023.

For manuscripts utilizing custom algorithms or software that are central to the research but not yet described in published literature, software must be made available to editors and reviewers. We strongly encourage code deposition in a community repository (e.g. GitHub). See the Nature Portfolio guidelines for submitting code & software for further information.

Data

Policy information about availability of data

All manuscripts must include a data availability statement. This statement should provide the following information, where applicable:

- Accession codes, unique identifiers, or web links for publicly available datasets
- A description of any restrictions on data availability
- For clinical datasets or third party data, please ensure that the statement adheres to our policy

To test the model, we digitized fossil diversity data from 2 published articles [Alroy J. Science 329, 1191–1194 (2010); Zaffos et al. PNAS 114, 5653–5658 (2017)] using the free software XYscan under the terms of the GNU General Public License as published by the Free Software Foundation. Copyright 2002-2021 Thomas S. Ullrich (https://rhig.physics.yale.edu/~ullrich/software/xyscan/). In addition, the Sepkoski's fossil diversity data were downloaded from the Sepkoski's Online Genus Database at the following link: http://strata.geology.wisc.edu/jack/. All these data are supplied as Source_Data_File1_FossilTimeSeries.xlsx through the Nature online submission system. These data were also used to calculate the magnitude, time and duration of mass extinctions (net diversification rates). The negative net diversification rates during mass extinctions are supplied as Source_Data_File2_mass_extinctions.xlsx files. Finally, modern estimates of marine invertebrate diversity were obtained from occurrence records of genera belonging to two of the most diverse groups of marine invertebrates, crustaceans and molluscs, as downloaded from the Ocean Biodiversity Information System (OBIS) database on 22nd October 2021 (www.obis.org). These diversity data are suplied with this paper as Source_Data_File3_OBIS_data.xlsx file.

The coupled paleogeographic-diversification model presented here uses input data of seafloor age distributions and paleoenvironmental conditions from the siwill22/agegrid-0.1 v1-alpha paleogeographic model and the cGENIE Earth System Model, respectively. We provide code availability for each of these two models. Additionally, all data inputs required to run the coupled paleogeographic-diversification model are available along with the model code on GitHub (https://github.com/CarmenGarciaComas/INDITEK).

Field-specific reporting

Please select the one below that is the best fit for your research. If you are not sure, read the appropriate sections before making your selection.

☐ Life sciences ☐ Behavioural & social sciences ☒ Ecological, evolutionary & environmental sciences

For a reference copy of the document with all sections, see nature.com/documents/nr-reporting-summary-flat.pdf

Life sciences study design

All studies must disclose on these points even when the disclosure is negative.

Sample size	*Describe how sample size was determined, detailing any statistical methods used to predetermine sample size OR if no sample-size calculation was performed, describe how sample sizes were chosen and provide a rationale for why these sample sizes are sufficient.*
Data exclusions	*Describe any data exclusions. If no data were excluded from the analyses, state so OR if data were excluded, describe the exclusions and the rationale behind them, indicating whether exclusion criteria were pre-established.*
Replication	*Describe the measures taken to verify the reproducibility of the experimental findings. If all attempts at replication were successful, confirm this OR if there are any findings that were not replicated or cannot be reproduced, note this and describe why.*
Randomization	*Describe how samples/organisms/participants were allocated into experimental groups. If allocation was not random, describe how covariates were controlled OR if this is not relevant to your study, explain why.*
Blinding	*Describe whether the investigators were blinded to group allocation during data collection and/or analysis. If blinding was not possible, describe why OR explain why blinding was not relevant to your study.*

Behavioural & social sciences study design

All studies must disclose on these points even when the disclosure is negative.

Study description	*Briefly describe the study type including whether data are quantitative, qualitative, or mixed-methods (e.g. qualitative cross-sectional, quantitative experimental, mixed-methods case study).*
Research sample	*State the research sample (e.g. Harvard university undergraduates, villagers in rural India) and provide relevant demographic information (e.g. age, sex) and indicate whether the sample is representative. Provide a rationale for the study sample chosen. For studies involving existing datasets, please describe the dataset and source.*
Sampling strategy	*Describe the sampling procedure (e.g. random, snowball, stratified, convenience). Describe the statistical methods that were used to predetermine sample size OR if no sample-size calculation was performed, describe how sample sizes were chosen and provide a rationale for why these sample sizes are sufficient. For qualitative data, please indicate whether data saturation was considered, and what criteria were used to decide that no further sampling was needed.*

Data collection	*Provide details about the data collection procedure, including the instruments or devices used to record the data (e.g. pen and paper, computer, eye tracker, video or audio equipment) whether anyone was present besides the participant(s) and the researcher, and whether the researcher was blind to experimental condition and/or the study hypothesis during data collection.*
Timing	*Indicate the start and stop dates of data collection. If there is a gap between collection periods, state the dates for each sample cohort.*
Data exclusions	*If no data were excluded from the analyses, state so OR if data were excluded, provide the exact number of exclusions and the rationale behind them, indicating whether exclusion criteria were pre-established.*
Non-participation	*State how many participants dropped out/declined participation and the reason(s) given OR provide response rate OR state that no participants dropped out/declined participation.*
Randomization	*If participants were not allocated into experimental groups, state so OR describe how participants were allocated to groups, and if allocation was not random, describe how covariates were controlled.*

Ecological, evolutionary & environmental sciences study design

All studies must disclose on these points even when the disclosure is negative.

Study description	We present a regional diversification model of marine animals for the Phanerozoic. The diversification model is coupled to a palaeogeographic model that constrains evolutionary time within regions (i.e. the age of the seafloor for deep ocean regions and the time underwater for flooded continental regions). An Earth System model provides palaeoenvironmental conditions (i.e. seawater temperature and organic C export fluxes, as a surrogate for food supply). The coupled model tracks the geographic position of ocean and flooded continental points every ca. 5 Myr, from 541 Ma to the present. Each and every one of the tracked points accumulate diversity over time at a different rate, which is modulated by the environmental history of each point. The model reproduces the present-day spatial distributions and fossil time-trajectories of marine invertebrate diversity after imposing mass extinctions. We find that the dynamics of global fossil diversity is best described by a diversification model that operates widely within the exponential growth regime of a logistic function. A spatially resolved analysis of the diversity-to-carrying capacity ratio reveals that <2% of the global flooded continental area throughout the Phanerozoic exhibits levels of diversity approaching ecological saturation. Our model corroborates earlier claims that Earth's environmental history and the patterns of continental fragmentation and reassembly have been major determinants of marine animal diversification. The analysis also shows that the development of diversity hotspots played a major role in the overall increase in global diversity during the late Mesozoic and Cenozoic.
Research sample	This is a modeling study. We test the model against three published fossil diversity curves. Fossil diversity curves are digitized from 2 published articles [Alroy J. Science 329, 1191–1194 (2010); Zaffos et al. PNAS 114, 5653–5658 (2017)] using the free software XYscan under the terms of the GNU General Public License as published by the Free Software Foundation. Copyright 2002-2021 Thomas S. Ullrich. In addition, the Sepkoski's fossil diversity data are downloaded from the Sepkoski's Online Genus Database at the following link: http://strata.geology.wisc.edu/jack/. Modern estimates of marine invertebrate diversity are downloaded from the Ocean Biodiversity Information System (OBIS) database on 22nd October 2021 (www.obis.org).
Sampling strategy	N/A - There is no sampling strategy as it is a model.
Data collection	The coupled paleogeographic-diversification model presented here uses input data of seafloor age distributions and paleoenvironmental conditions from the siwill22/agegrid-0.1 v1-alpha paleogeographic model (DOI: 10.5281/zenodo.3271360) and the cGENIE Earth System Model (DOI: 10.5281/zenodo.4618023), respectively. Additionally, all data inputs required to run the coupled paleogeographic-diversification model are available along with the model code on GitHub (https://github.com/ CarmenGarciaComas/INDITEK).<br><br>To compare our data outputs with observations, we scanned published fossil curves and downloaded observations of taxa from a global dataset of marine diversity. Digitized fossil diversity data from the Alroy and Zaffos et al. published articles with the free software XYscan (https://rhig.physics.yale.edu/~ullrich/software/xyscan/) and those downloaded from the Sepkoski's Online Genus Database (http://strata.geology.wisc.edu/jack/) are provided as Source data with this paper: Source_Data_File1_FossilTimeSeries.xlsx. Diversity data downloaded from the Ocean Biodiversity Information System (OBIS) database on 22nd October 2021 (www.obis.org) are also provided as Source data with this paper: Source_Data_File3_OBIS_data.xlsx.
Timing and spatial scale	This is a modeling study and therefore there is no data collection. The model analysis covers the global ocean for the last 541 My. The palaeogeographic model mimics plate tectonics and traces the age and position of thousands of seafloor points (for the deep ocean and for the flooded continental regions) in 82 time frames about every 5 My (exact time frames: 541 535 525 515 510 505 500 495 490 485 480 475 470 465 455 450 445 440 435 430 425 420 415 410 400 390 385 375 365 355 340 325 320 310 305 300 295 285 275 270 265 260 255 250 245 240 230 220 205 200 195 185 180 170 165 160 155 150 140 135 130 125 120 105 95 90 85 80 70 65 60 50 45 40 35 30 25 20 15 10 5 0 Mya). Time frames were selected as representative of tectonic changes around those dates with computation limitation. The Earth system model provides paleoenvironmental conditions (seawater temperature and deep-time food supply) with a spatio-temporal resolution of 36x36 equal-area grids every 20 My (exact time frames: 541 530 520 510 496 436 415 400 366 327 301 265 234 196 178 168 149 131 116 107 97 87 75 69 61 52 36 26 15 0 Mya). Time frames and spatial resolution were selected as representative of tectonic changes around those dates with computation limitation. The paleoenvironmental conditions were interpolated into the seafloor points for the 82 time frames in a 2-step fashion (see Methods for details). Comparison of model outputs with fossil curves was done for the 82 time frames by scanning the fossil time series. Spatial comparison of 0 Mya with current diversity was done for 0.5x0.5 grid maps and mean latitudinal values of OBIS data after correcting for sampling effort (see Methods for details).

Data exclusions	This is a modeling study and we only use data (i.e. fossil diversity curves and observations of marine invertebrates extracted from the OBIS database) to test the global diversity time series and modern spatial diversity distributions generated by the model. To calculate Lin's concordance correlation coefficient, which provides an estimate of how much the global diversity curves in our model differ from the fossil diversity curves, we exclude data points that are within mass extinction intervals. The reasoning is that mass extinctions are imposed externally on the model and, therefore, leaving them would inflate the statistical fits. For the comparison with OBIS data, we focus on continental margins, which harbour the vast majority of the diversity of marine invertebrates.
Reproducibility	This is a modeling study. All the analysis and results can be replicated using the model code that is available on GitHub (https://github.com/CarmenGarciaComas/INDITEK). The results are consistently repeated using 40 different combinations of model parameters. Furthermore, we impose extinction patterns extracted from 3 different (published) fossil diversity curves and test the model against all of them. In addition, we test the robustness of the model against various configurations of the paleogeographic model and the earth system by conducting a series of sensitivity analyses. Sensitivities to changes in the palaeogeographic reconstruction models, sea level, or the oceanic inventory of phosphorus over the past 541 million years. In all cases, the results and conclusions of the study remain the same.
Randomization	N/A as data were not issued from a sampling strategy. This is a modeling study and the results are generated from the simulations that result from running the model.
Blinding	N/A as data were not issued from a sampling strategy.

Did the study involve field work? ☐ Yes ☒ No

Field work, collection and transport

Field conditions	N/A
Location	N/A
Access & import/export	N/A
Disturbance	N/A

Reporting for specific materials, systems and methods

We require information from authors about some types of materials, experimental systems and methods used in many studies. Here, indicate whether each material, system or method listed is relevant to your study. If you are not sure if a list item applies to your research, read the appropriate section before selecting a response.

Materials & experimental systems

n/a	Involved in the study	
☒	☐	Antibodies
☒	☐	Eukaryotic cell lines
☒	☐	Palaeontology and archaeology
☒	☐	Animals and other organisms
☒	☐	Human research participants
☒	☐	Clinical data
☒	☐	Dual use research of concern

Methods

n/a	Involved in the study	
☒	☐	ChIP-seq
☒	☐	Flow cytometry
☒	☐	MRI-based neuroimaging

Antibodies

Antibodies used	*Describe all antibodies used in the study; as applicable, provide supplier name, catalog number, clone name, and lot number.*
Validation	*Describe the validation of each primary antibody for the species and application, noting any validation statements on the manufacturer's website, relevant citations, antibody profiles in online databases, or data provided in the manuscript.*

Eukaryotic cell lines

Policy information about cell lines

Cell line source(s)	*State the source of each cell line used.*
Authentication	*Describe the authentication procedures for each cell line used OR declare that none of the cell lines used were authenticated.*
Mycoplasma contamination	*Confirm that all cell lines tested negative for mycoplasma contamination OR describe the results of the testing for mycoplasma contamination OR declare that the cell lines were not tested for mycoplasma contamination.*

Commonly misidentified lines (See ICLAC register) | *Name any commonly misidentified cell lines used in the study and provide a rationale for their use.*

Palaeontology and Archaeology

Specimen provenance | *Provide provenance information for specimens and describe permits that were obtained for the work (including the name of the issuing authority, the date of issue, and any identifying information). Permits should encompass collection and, where applicable, export.*

Specimen deposition | *Indicate where the specimens have been deposited to permit free access by other researchers.*

Dating methods | *If new dates are provided, describe how they were obtained (e.g. collection, storage, sample pretreatment and measurement), where they were obtained (i.e. lab name), the calibration program and the protocol for quality assurance OR state that no new dates are provided.*

☐ Tick this box to confirm that the raw and calibrated dates are available in the paper or in Supplementary Information.

Ethics oversight | *Identify the organization(s) that approved or provided guidance on the study protocol, OR state that no ethical approval or guidance was required and explain why not.*

Note that full information on the approval of the study protocol must also be provided in the manuscript.

Animals and other organisms

Policy information about studies involving animals; ARRIVE guidelines recommended for reporting animal research

Laboratory animals | *For laboratory animals, report species, strain, sex and age OR state that the study did not involve laboratory animals.*

Wild animals | *Provide details on animals observed in or captured in the field; report species, sex and age where possible. Describe how animals were caught and transported and what happened to captive animals after the study (if killed, explain why and describe method; if released, say where and when) OR state that the study did not involve wild animals.*

Field-collected samples | *For laboratory work with field-collected samples, describe all relevant parameters such as housing, maintenance, temperature, photoperiod and end-of-experiment protocol OR state that the study did not involve samples collected from the field.*

Ethics oversight | *Identify the organization(s) that approved or provided guidance on the study protocol, OR state that no ethical approval or guidance was required and explain why not.*

Note that full information on the approval of the study protocol must also be provided in the manuscript.

Human research participants

Policy information about studies involving human research participants

Population characteristics | *Describe the covariate-relevant population characteristics of the human research participants (e.g. age, gender, genotypic information, past and current diagnosis and treatment categories). If you filled out the behavioural & social sciences study design questions and have nothing to add here, write "See above."*

Recruitment | *Describe how participants were recruited. Outline any potential self-selection bias or other biases that may be present and how these are likely to impact results.*

Ethics oversight | *Identify the organization(s) that approved the study protocol.*

Note that full information on the approval of the study protocol must also be provided in the manuscript.

Clinical data

Policy information about clinical studies

All manuscripts should comply with the ICMJE guidelines for publication of clinical research and a completed CONSORT checklist must be included with all submissions.

Clinical trial registration | *Provide the trial registration number from ClinicalTrials.gov or an equivalent agency.*

Study protocol | *Note where the full trial protocol can be accessed OR if not available, explain why.*

Data collection | *Describe the settings and locales of data collection, noting the time periods of recruitment and data collection.*

Outcomes | *Describe how you pre-defined primary and secondary outcome measures and how you assessed these measures.*

Dual use research of concern

Policy information about dual use research of concern

Hazards

Could the accidental, deliberate or reckless misuse of agents or technologies generated in the work, or the application of information presented in the manuscript, pose a threat to:

No	Yes	
☐	☐	Public health
☐	☐	National security
☐	☐	Crops and/or livestock
☐	☐	Ecosystems
☐	☐	Any other significant area

Experiments of concern

Does the work involve any of these experiments of concern:

No	Yes	
☐	☐	Demonstrate how to render a vaccine ineffective
☐	☐	Confer resistance to therapeutically useful antibiotics or antiviral agents
☐	☐	Enhance the virulence of a pathogen or render a nonpathogen virulent
☐	☐	Increase transmissibility of a pathogen
☐	☐	Alter the host range of a pathogen
☐	☐	Enable evasion of diagnostic/detection modalities
☐	☐	Enable the weaponization of a biological agent or toxin
☐	☐	Any other potentially harmful combination of experiments and agents

ChIP-seq

Data deposition

☐ Confirm that both raw and final processed data have been deposited in a public database such as GEO.

☐ Confirm that you have deposited or provided access to graph files (e.g. BED files) for the called peaks.

Data access links<br>*May remain private before publication.*	*For "Initial submission" or "Revised version" documents, provide reviewer access links. For your "Final submission" document, provide a link to the deposited data.*
Files in database submission	*Provide a list of all files available in the database submission.*
Genome browser session<br>(e.g. UCSC)	*Provide a link to an anonymized genome browser session for "Initial submission" and "Revised version" documents only, to enable peer review. Write "no longer applicable" for "Final submission" documents.*

Methodology

Replicates	*Describe the experimental replicates, specifying number, type and replicate agreement.*
Sequencing depth	*Describe the sequencing depth for each experiment, providing the total number of reads, uniquely mapped reads, length of reads and whether they were paired- or single-end.*
Antibodies	*Describe the antibodies used for the ChIP-seq experiments; as applicable, provide supplier name, catalog number, clone name, and lot number.*
Peak calling parameters	*Specify the command line program and parameters used for read mapping and peak calling, including the ChIP, control and index files used.*
Data quality	*Describe the methods used to ensure data quality in full detail, including how many peaks are at FDR 5% and above 5-fold enrichment.*
Software	*Describe the software used to collect and analyze the ChIP-seq data. For custom code that has been deposited into a community repository, provide accession details.*

Flow Cytometry

Plots

Confirm that:

- [ ] The axis labels state the marker and fluorochrome used (e.g. CD4-FITC).
- [ ] The axis scales are clearly visible. Include numbers along axes only for bottom left plot of group (a 'group' is an analysis of identical markers).
- [ ] All plots are contour plots with outliers or pseudocolor plots.
- [ ] A numerical value for number of cells or percentage (with statistics) is provided.

Methodology

Sample preparation	*Describe the sample preparation, detailing the biological source of the cells and any tissue processing steps used.*
Instrument	*Identify the instrument used for data collection, specifying make and model number.*
Software	*Describe the software used to collect and analyze the flow cytometry data. For custom code that has been deposited into a community repository, provide accession details.*
Cell population abundance	*Describe the abundance of the relevant cell populations within post-sort fractions, providing details on the purity of the samples and how it was determined.*
Gating strategy	*Describe the gating strategy used for all relevant experiments, specifying the preliminary FSC/SSC gates of the starting cell population, indicating where boundaries between "positive" and "negative" staining cell populations are defined.*

- [ ] Tick this box to confirm that a figure exemplifying the gating strategy is provided in the Supplementary Information.

Magnetic resonance imaging

Experimental design

Design type	*Indicate task or resting state; event-related or block design.*
Design specifications	*Specify the number of blocks, trials or experimental units per session and/or subject, and specify the length of each trial or block (if trials are blocked) and interval between trials.*
Behavioral performance measures	*State number and/or type of variables recorded (e.g. correct button press, response time) and what statistics were used to establish that the subjects were performing the task as expected (e.g. mean, range, and/or standard deviation across subjects).*

Acquisition

Imaging type(s)	*Specify: functional, structural, diffusion, perfusion.*
Field strength	*Specify in Tesla*
Sequence & imaging parameters	*Specify the pulse sequence type (gradient echo, spin echo, etc.), imaging type (EPI, spiral, etc.), field of view, matrix size, slice thickness, orientation and TE/TR/flip angle.*
Area of acquisition	*State whether a whole brain scan was used OR define the area of acquisition, describing how the region was determined.*

Diffusion MRI ☐ Used ☐ Not used

Preprocessing

Preprocessing software	*Provide detail on software version and revision number and on specific parameters (model/functions, brain extraction, segmentation, smoothing kernel size, etc.).*
Normalization	*If data were normalized/standardized, describe the approach(es): specify linear or non-linear and define image types used for transformation OR indicate that data were not normalized and explain rationale for lack of normalization.*
Normalization template	*Describe the template used for normalization/transformation, specifying subject space or group standardized space (e.g. original Talairach, MNI305, ICBM152) OR indicate that the data were not normalized.*
Noise and artifact removal	*Describe your procedure(s) for artifact and structured noise removal, specifying motion parameters, tissue signals and physiological signals (heart rate, respiration).*

Volume censoring | *Define your software and/or method and criteria for volume censoring, and state the extent of such censoring.*

Statistical modeling & inference

Model type and settings | *Specify type (mass univariate, multivariate, RSA, predictive, etc.) and describe essential details of the model at the first and second levels (e.g. fixed, random or mixed effects; drift or auto-correlation).*

Effect(s) tested | *Define precise effect in terms of the task or stimulus conditions instead of psychological concepts and indicate whether ANOVA or factorial designs were used.*

Specify type of analysis: ☐ Whole brain ☐ ROI-based ☐ Both

Statistic type for inference
(See Eklund et al. 2016) | *Specify voxel-wise or cluster-wise and report all relevant parameters for cluster-wise methods.*

Correction | *Describe the type of correction and how it is obtained for multiple comparisons (e.g. FWE, FDR, permutation or Monte Carlo).*

Models & analysis

n/a	Involved in the study
☐	☐ Functional and/or effective connectivity
☐	☐ Graph analysis
☐	☐ Multivariate modeling or predictive analysis

Functional and/or effective connectivity | *Report the measures of dependence used and the model details (e.g. Pearson correlation, partial correlation, mutual information).*

Graph analysis | *Report the dependent variable and connectivity measure, specifying weighted graph or binarized graph, subject- or group-level, and the global and/or node summaries used (e.g. clustering coefficient, efficiency, etc.).*

Multivariate modeling and predictive analysis | *Specify independent variables, features extraction and dimension reduction, model, training and evaluation metrics.*
